# Field output correction factors using a scintillation detector

**DOI:** 10.1002/mp.17729

**Published:** 2025-03-08

**Authors:** Luc Gingras, Yunuen Cervantes, Frederic Beaulieu, Magali Besnier, Benjamin Coté, Simon Lambert‐Girard, Danahé LeBlanc, Yoan LeChasseur, François Therriault‐Proulx, Luc Beaulieu, Louis Archambault

**Affiliations:** ^1^ Service de physique médicale et de radioprotection, Centre intégré de cancérologie CHU de Québec‐Université Laval et Centre de recherche du CHU de Québec Québec Québec Canada; ^2^ Département de physique, de génie physique et d'optique, et Centre de recherche sur le cancer Université Laval Québec Québec Canada; ^3^ Medscint Inc. Québec QC Canada

**Keywords:** dosimetry, external beam radiotherapy, ion chambers, output factors, plastic scintillation detectors, small field, solid state detectors

## Abstract

**Background:**

As small radiation fields play an ever‐increasing role in radiation therapy, accurate dosimetry of these fields becomes critical to ensure high quality dose calculation and treatment optimization. Despite the availability of several small volume dose detectors, small field dosimetry remains challenging. The PRB‐0002, a new plastic scintillation detector part of the Hyperscint RP‐200 dosimetric platform from Medscint, that requires only minimal corrections can potentially facilitate small field measurements.

**Purpose:**

The main objective of this work is to adapt small field formalism to plastic scintillation dosimetry using both Monte Carlo (MC) simulations and measurements. The secondary objective is to use the fully characterized PRB‐0002 for accurate and precise measurements of field output correction factors and compare those measurements with that from other small field detectors.

**Methods:**

Our work is based on IAEA TRS‐483 report. EGSnrc MC simulations of the PRB‐0002 were conducted to determine the impact of detector composition, surrounding materials, dose averaging within the sensitive volume as well as ionization quenching. From these simulations, the field output correction factors of PRB‐0002 were determined. Then, by experimental comparisons, field output correction factors for 2 solid state detectors and 3 small volume ion chambers have been determined.

**Results:**

With PRB‐0002, the material composition factor is well balanced with the ionization quenching making the field output correction factor near unity. For fields between 0.6 × 0.6 and 30 × 30 ${\mathrm{cm}}^{2}$, the field output correction factors of the PRB‐0002 were between 1.002 and 0.999 with a total uncertainty of 0.5%. Analysis of the uncertainty budget showed that, using PRB‐0002 for measuring output factors an overall uncertainty of 0.59% can be achieved for a 1 × 1 ${\mathrm{cm}}^{2}$ field size.

**Conclusions:**

With field output correction factors close to unity for a wide range of field sizes, the PRB‐0002 is a near‐ideal detector for small field dosimetry. Furthermore, it can be used to experimentally determine the field output correction factors of other dosimeters with great accuracy.

## 1 INTRODUCTION

Radiation therapy (RT) has evolved from treating large anatomical areas encompassing both the cancer and copious amount of healthy tissues to delivering dose distributions that can precisely match the shape of a tumor. This evolution has been driven by the increased mechanical capabilities of medical linear accelerators (linacs) and by the widespread availability of imaging modalities (MRI, CT, CBCT) prior to and during treatment. The underlying hypothesis behind modern RT is that treating smaller volumes makes it possible to maximize the dose to the cancer while minimizing damage to healthy tissues thus achieving better clinical outcomes.

Treating small volumes necessarily involves at least some small radiation fields. Indeed, most advanced treatment techniques such as intensity modulated radiation therapy, volumetric modulated arc therapy and stereotactic radiosurgery rely heavily on small fields. To guarantee the accuracy of these treatments, it is of a prime importance that small fields are well characterized and well modeled in treatment planning systems.^1^

Dosimetry of small fields has been studied for decades either directly or through the broader categories of non‐equilibrium^2^ or non‐standard^3^ fields. Today, the most accurate definition is provided by the IAEA‐AAPM TRS‐483 code of practice^4^: a field is considered small if at least one of the following conditions is met: (1) a loss of lateral charged particle equilibrium on the beam axis; (2) a partial occlusion of the primary photon source by the collimating device on the beam axis; (3) the size of the detector is similar or larger than the beam dimensions. While the conditions 1 and 2 are directly related to the beam, condition 3 depends on the detector used to perform a measurement. Thus, a field might be considered small for one detector and not for another.

The challenges of small field dosimetry are well documented (see refs. [3, 4] for a good summary). According to TRS‐483, the increased usage of small fields “*has increased the uncertainty of clinical dosimetry and weakened its traceability to reference dosimetry based on conventional [codes of practice]*”.^4^ It is in this context that the IAEA‐AAPM proposed the TRS‐483 code of practice to provide a standardized method for the determination of field output factors for relative dosimetry in small static photon fields.

A field output factor, Ω, is the ratio of the dose to water at the center of two static fields: a clinical field, $f_{\text{clin}}$ (of quality $Q_{\text{clin}}$) and a reference field, either a standard reference, $f_{\text{ref}}$ (of quality Q), or a machine specific reference, $f_{\text{msr}}$ (of quality $Q_{\text{msr}}$). For large field sizes, the dose ratio is well approximated by the ratio of measured dosimeter signals, because the conversion from a detector measurement to a dose to water is mostly independent of field size. However, for small fields, this conversion depends on the field size because of the volume averaging, the perturbation of the charged particle fluence due to the presence of the detector and the intrinsic change in beam quality with field size. A field output correction factor, $k_{Q_{\mathrm{clin}},Q_{\mathrm{msr}}}^{f_{\mathrm{clin}},f_{\mathrm{msr}}}$ must therefore be applied to the ratio of measurements in order to determine Ω of small fields.

Accurate determination of $k_{Q_{\mathrm{clin}},Q_{\mathrm{msr}}}^{f_{\mathrm{clin}},f_{\mathrm{msr}}}$ is one of the main challenges of small field dosimetry. Values are tabulated in TRS‐483^4^ for a wide range of detectors. These data, taken from the literature, consider both experimental measurements and Monte Carlo (MC) simulations. Nevertheless, it might be necessary to determine $k_{Q_{\mathrm{clin}},Q_{\mathrm{msr}}}^{f_{\mathrm{clin}},f_{\mathrm{msr}}}$ for detectors or beam quality not listed^5^, ^6^, ^7^ or at higher accuracy level, for detectors in different orientations^8^ or to perform an independent evaluation of tabulated values. In their initial report, Alfonso et al.^3^ mentioned calculating this factor with MC simulation alone or measuring it with a *suitable*, well characterized detector.

The search for an ideal reference detector for small field dosimetry is ongoing. The list of properties for such a detector is well known (see tab. 6 of ref. [4]). Some examples include: stability, spatial resolution, dose, and dose rate linearity. Also, in general, a good reference detector should have an $k_{Q_{\mathrm{clin}},Q_{\mathrm{msr}}}^{f_{\mathrm{clin}},f_{\mathrm{msr}}}$ as close to 1 as possible. Because of their large correction factors, ion chambers (including small‐volume ion chambers) are usually not ideal for reference dosimetry of small fields, despite being the gold standard for other types of beams. Diodes, diamond detector, radiochromic films, plastic scintillation detectors (PSDs) and alanine dosimeters have all been investigated as potential candidates for reference measurements, but none of them has emerged as clear choice.

PSDs possess many advantages for small field dosimetry^9^, ^10^ and are generally thought to have an output correction factor close to unity.^4^ Because of these properties, Casar et al.^6^ have recently used a PSD as a reference detector to determine the $k_{Q_{\mathrm{clin}},Q_{\mathrm{msr}}}^{f_{\mathrm{clin}},f_{\mathrm{msr}}}$ of several other detectors.

In a PSD, the scintillation signal is usually assumed to be proportional to the dose received^11^ although PSDs are known to suffer from ionization quenching (i.e., a diminution of the scintillation light emitted per unit dose) when irradiated by beams of particles of high linear energy transfer like protons and other hadrons.^12^ It has been recently shown by Santurio et al.^13^, ^14^ that ionization quenching can also be observed in megavoltage photon beams because of the presence of low energy secondary electrons. This means that the signal produced by a PSD can depend on beam quality and field size. To account for this, the ionization quenching correction factor, $k_{\text{ioq}}$ was introduced^13^ and determined using MC simulations. Because of this factor, the overall output correction factor of a PSD could differ from unity.

In this context, the new PRB‐0002 PSD has been developed by Medscint (Medscint inc., Quebec city, Canada) and previously characterized.^15^ The scintillator probe, a component of the Hyperscint RP‐200 dosimetry platform, was designed to have a $k_{Q_{\mathrm{clin}},Q_{\mathrm{msr}}}^{f_{\mathrm{clin}},f_{\mathrm{msr}}}$ as close to 1 as possible even when including the impact of $k_{\text{ioq}}$. The goal of this work is (1) to determine the output correction factor of this new probe using MC simulations and then (2) to use it as a reference in order to determine the $k_{Q_{\mathrm{clin}},Q_{\mathrm{msr}}}^{f_{\mathrm{clin}},f_{\mathrm{msr}}}$ of other detectors for small fields.

## 2 METHODS AND MATERIALS

### 2.1 Formalism and definitions

Following the methodology of the TG‐51 report,^16^ the absorbed dose to water of a field f of quality Q, noted $D_{w,Q}^{f}$, can be calculated from a detector reading corrected for environmental conditions, signal collection effects and positional uncertainty, and which is noted $M_{Q}^{f}$. For dosimetry with an ion chamber detector in a clinical field (clin),^3^ the dose to water in the absence of the detector is given by

(1) $$D_{w,Q_{\mathrm{clin}}}^{f_{\mathrm{clin}}}=M_{Q_{\mathrm{clin}}}^{f_{\mathrm{clin}}}N_{D,w,Q_{0}}^{f_{\mathrm{ref}}}k_{Q_{\mathrm{msr}},Q_{0}}^{f_{\mathrm{msr}},f_{\mathrm{ref}}}k_{Q_{\mathrm{clin}},Q_{\mathrm{msr}}}^{f_{\mathrm{clin}},f_{\mathrm{msr}}},$$

where the reference class ionization chamber (IC) calibration coefficient $N_{D,w,Q_{0}}^{f_{\mathrm{ref}}}$ is available from a standards laboratory's reference beam of quality $Q_{0}$ in the conventional reference field $f_{\mathrm{ref}}$. The measured detector reading for a given clinical field, $M_{Q_{\mathrm{clin}}}^{f_{\mathrm{clin}}}$ is corrected first by a factor $k_{Q_{\mathrm{clin}},Q_{\mathrm{msr}}}^{f_{\mathrm{clin}},f_{\mathrm{msr}}}$ to account for the difference in beam quality between $\left(f_{\mathrm{clin}},Q_{\mathrm{clin}}\right)$ and the machine specific reference $\left(f_{\mathrm{msr}},Q_{\mathrm{msr}}\right)$ and then by a factor $k_{Q_{\mathrm{msr}},Q_{0}}^{f_{\mathrm{msr}},f_{\mathrm{ref}}}$, to account for the difference in beam quality between the reference condition $\left(f_{\mathrm{ref}},Q_{0}\right)$ and the machine specific reference.

Based on the formalism of Alfonso et al.,^3^ the field output factor $\Omega_{Q_{\mathrm{clin}},Q_{\mathrm{msr}}}^{f_{\mathrm{clin}},f_{\mathrm{msr}}}$ can be determined using Equations (17) and (18) of TRS‐483^4^

(2) $$\Omega_{Q_{\mathrm{clin}},Q_{\mathrm{msr}}}^{f_{\mathrm{clin}},f_{\mathrm{msr}}}=\frac{D_{w,Q_{\mathrm{clin}}}^{f_{\mathrm{clin}}}}{D_{w,Q_{\mathrm{msr}}}^{f_{\mathrm{msr}}}}=\frac{M_{Q_{\mathrm{clin}}}^{f_{\mathrm{clin}}}}{M_{Q_{\mathrm{msr}}}^{f_{\mathrm{msr}}}}k_{Q_{\mathrm{clin}},Q_{\mathrm{msr}}}^{f_{\mathrm{clin}},f_{\mathrm{msr}}},$$

In order to determine the field output factor from a measurement ratio, it is necessary to know the detector specific output correction factors, $k_{Q_{\mathrm{clin}},Q_{\mathrm{msr}}}^{f_{\mathrm{clin}},f_{\mathrm{msr}}}$. From Equation (2), we can write

(3) $$k_{Q_{\mathrm{clin}},Q_{\mathrm{msr}}}^{f_{\mathrm{clin}},f_{\mathrm{msr}}}=\frac{\left(D_{w,Q_{\mathrm{clin}}}^{f_{\mathrm{clin}}}/D_{w,Q_{\mathrm{msr}}}^{f_{\mathrm{msr}}}\right)}{\left(M_{Q_{\mathrm{clin}}}^{f_{\mathrm{clin}}}/M_{Q_{\mathrm{msr}}}^{f_{\mathrm{msr}}}\right)},$$

As detailed in Appendix II of TRS‐483,^4^ this correction factor can be obtained by three approaches: (i) using a perturbation free (except for volume averaging) reference detector to obtain the dose ratio in Equation (3); (ii) using a reference detector with known output correction factor to evaluate the same ratio; and (iii) using MC simulations to directly determine Equation (3).

The first method has been used with “perturbation free” reference detectors such as alanine, TLDs, organic scintillators, and radiochromic films.^6^, ^17^ The second method has been used to characterize detectors using a another detector that is well characterized at the same irradiation conditions.^6^, ^18^ Assuming that the output correction factor is known for detector 1 (det1), this correction can be determined for detector 2 (det2) using Equation (2)

(4) $${\left[k_{Q_{\mathrm{clin}},Q_{\mathrm{msr}}}^{f_{\mathrm{clin}},f_{\mathrm{msr}}}\right]}_{\det_{2}}={\left[\frac{M_{Q_{\mathrm{clin}}}^{f_{\mathrm{clin}}}}{M_{Q_{\mathrm{msr}}}^{f_{\mathrm{msr}}}}k_{Q_{\mathrm{clin}},Q_{\mathrm{msr}}}^{f_{\mathrm{clin}},f_{\mathrm{msr}}}\right]}_{\det_{1}}{\left[\frac{M_{Q_{\mathrm{msr}}}^{f_{\mathrm{msr}}}}{M_{Q_{\mathrm{clin}}}^{f_{\mathrm{clin}}}}\right]}_{\det_{2}}.$$

The third method involves the use of MC calculations simulating both the radiation source with its collimation device and the irradiated phantom medium. Simulation material can be either water for the $D_{Q}^{f}$ ratio or complete detector material and geometry in water for the $M_{Q}^{f}$ ratio in Equation (3). A hybrid method that uses MC simulations for $D_{Q}^{f}$ ratio and detector measurements for $M_{Q}^{f}$ ratio has also been proposed.^19^ However, such hybrid approach is sensitive to the accuracy of the radiation source model because particle energy fluence distribution may be different between the simulation and measurement.

While the signal of an ideal dosimeter should only be proportional to the average dose deposited in its sensitive volume (${\bar{D}}_{\det,Q}^{f}$), most detectors exhibit some dependency to other factors. The signal production per unit dose may depend on environmental factors, operating conditions, beam quality, as well as temporal dynamics of dose delivery. Signal collection can also be affected by operating conditions and detector‐reader coupling configuration. Factors affecting signal production and collection are discussed in length in TG‐51 and TRS‐483.^4^, ^16^

We distinguish two types of correction factors that can be applied to raw measurement signals ($M_{\mathrm{raw}}$) of a detector to transform it into a quantity solely dependent on the dose absorbed by its sensitive volume. The first type (I) includes corrections for well known influence quantities (e.g., temperature, pressure) that are independent of small changes in beam quality. In other words, the value of a type I correction factor should not change when going from a beam of quality $Q_{\mathrm{msr}}$ to a beam of quality $Q_{\mathrm{clin}}$. The second type (II) includes corrections for influence quantities that may vary with small changes in beam quality. This can be represented by the following equations:

(5) $$\begin{array}{cc} {\bar{D}}_{\det,Q}^{f} & \propto {\left(M_{\mathrm{raw}}\right)}_{Q}^{f}\times \prod_{i}{\left(k_{I,i}\right)}_{Q}^{f}\times \prod_{j}{\left(k_{II,j}\right)}_{Q}^{f}, \end{array}$$

(6) $$\begin{array}{cc} {\bar{D}}_{\det,Q}^{f} & \propto M_{Q}^{f}\times \prod_{j}{\left(k_{II,j}\right)}_{Q}^{f}, \end{array}$$

where ${\left(M_{raw}\right)}_{Q}^{f}$ and $M_{Q}^{f}$ are respectively the raw measurement signal and the measurement signal corrected for all influence quantities independent of small changes in beam quality. *Small changes* in beam quality are assumed to be changes that can easily occur in a measurement sequence. For example, changes in Q caused by varying the field size of a photon beam. From Equation (6), we can express the ratio of measurements corrected for type I factors

(7) $$\frac{M_{Q_{\mathrm{clin}}}^{f_{\mathrm{clin}}}}{M_{Q_{\mathrm{msr}}}^{f_{\mathrm{msr}}}}=\left[\frac{{\bar{D}}_{\det,Q_{\mathrm{clin}}}^{f_{\mathrm{clin}}}}{{\bar{D}}_{\det,Q_{\mathrm{msr}}}^{f_{\mathrm{msr}}}}\right]\times \left[\frac{1}{\prod_{j}{\left(k_{II,j}\right)}_{Q_{\mathrm{clin}},Q_{\mathrm{msr}}}^{f_{\mathrm{clin}},f_{\mathrm{msr}}}}\right],$$

Combining Equations (7) and (3)

(8) $$k_{Q_{\mathrm{clin}},Q_{\mathrm{msr}}}^{f_{\mathrm{clin}},f_{\mathrm{msr}}}=\left[\frac{\frac{D_{w,Q_{\mathrm{clin}}}^{f_{\mathrm{clin}}}}{D_{w,Q_{\mathrm{msr}}}^{f_{\mathrm{msr}}}}}{\frac{{\bar{D}}_{det,Q_{\mathrm{clin}}}^{f_{\mathrm{clin}}}}{{\bar{D}}_{det,Q_{\mathrm{msr}}}^{f_{\mathrm{msr}}}}}\right]\times \left[\prod_{j}{\left(k_{II,j}\right)}_{Q_{\mathrm{clin}},Q_{\mathrm{msr}}}^{f_{\mathrm{clin}},f_{\mathrm{msr}}}\right],$$

Thus, $k_{Q_{\mathrm{clin}},Q_{\mathrm{msr}}}^{f_{\mathrm{clin}},f_{\mathrm{msr}}}$ determination should take into account differences in type II factors between $Q_{\mathrm{clin}}$ and $Q_{\mathrm{msr}}$ using Equation (7). Most MC studies aiming to determine $k_{Q_{\mathrm{clin}},Q_{\mathrm{msr}}}^{f_{\mathrm{clin}},f_{\mathrm{msr}}}$ will implicitly assume type II corrections to be unity. While this is reasonable for ICs and solid state (SS) detectors in normal clinical conditions, it is not generally true for all detectors and conditions. For example, ionization quenching in PSDs should be accounted for by type II corrections. To compare $k_{Q_{\mathrm{clin}},Q_{\mathrm{msr}}}^{f_{\mathrm{clin}},f_{\mathrm{msr}}}$ values extracted from different studies, it is imperative to make sure that all non negligible type I measurement corrections have been applied in the same way on experimental data and that MC $k_{Q_{\mathrm{clin}},Q_{\mathrm{msr}}}^{f_{\mathrm{clin}},f_{\mathrm{msr}}}$ estimations include type II corrections if needed.

In this work, all experimental measurements were performed on a conventional linear accelerator (linac) capable of delivering a reference (ref) 10×10 ${\mathrm{cm}}^{2}$ field. Thus, in this case the machine specific reference (msr) and ref are identical conditions.

### 2.2 Measurement corrections

For IC, SS, and PSDs, the type I measurement correction equations take the following forms:

(9) $$\begin{array}{cc} M^{\mathrm{IC}} & =M_{\mathrm{raw}}^{\mathrm{IC}}k_{\mathrm{TP}}k_{H}k_{\mathrm{elec}}k_{\mathrm{pol}}k_{\mathrm{ion}}k_{\mathrm{drift}}k_{\mathrm{bg}}k_{\mathrm{stem}}k_{\mathrm{pos}} \end{array}$$

(10) $$\begin{array}{cc} M^{\mathrm{SS}} & =M_{\mathrm{raw}}^{\mathrm{SS}}k_{T}k_{\mathrm{elec}}k_{\mathrm{ion}}k_{\mathrm{drift}}k_{\mathrm{bg}}k_{\mathrm{stem}}k_{\mathrm{pos}} \end{array}$$

(11) $$\begin{array}{cc} M^{\mathrm{PSD}} & =M_{\mathrm{raw}}^{\mathrm{PSD}}k_{T}k_{\mathrm{read}}k_{\mathrm{drift}}k_{\mathrm{bg}}k_{\mathrm{stem}}k_{\mathrm{pos}} \end{array}$$

Most of these factors are well established in the literature; summarized definitions can be found in Supplementary Material 1.

All measurements conducted in this study are relative measurements between a clin field and a msr field. Thus, the only factors that need to be considered are those that change with field size or otherwise vary between these two measurement conditions. Reference field measurements were repeated frequently to ensure that drifts were small and could be taken into account when performing measurement ratios. Type I correction factor ratios ${\left(k_{I,i}\right)}^{f_{\mathrm{clin}},f_{\mathrm{msr}}}$, involving temperature, pressure, humidity, readout/electrometer and machine output drifts were taken as unity. Appropriate background subtraction also allowed use of $k_{bg}=1$. Furthermore, $k_{stem}^{f_{\mathrm{clin}},f_{\mathrm{msr}}}$ ratios were taken as unity, by selecting an appropriate field/detector orientation geometry. Uncertainties on ratios assumed to be unity were accounted for, but without a systematic component. Polarity, ionization recombination as well as positional variations were all considered because of their field size dependence. For ICs, $k_{\mathrm{pol}}$ was determined for each field size by averaging absolute positive and negative polarity raw measurements and dividing by the raw measurement at the normal operating polarity. After each polarity reversal, a pre‐irradiation of at least 8 Gy was applied to guarantee a stable detector output.

$k_{\mathrm{ion}}$ correction factors for ICs were determined using the TG‐51 addendum^20^ including a constant initial recombination term and a detector dose per pulse dependant general recombination term. Using a development similar to that of Duchaine et al.,^21^ it is possible to obtain an expression for $k_{\mathrm{ion}}$ ratio

(12) $${\left(k_{\mathrm{ion}}\right)}_{Q_{\mathrm{clin}},Q_{\mathrm{msr}}}^{f_{\mathrm{clin}},f_{\mathrm{msr}}}=\frac{1}{1+B\left(1-{\left(M_{\mathrm{raw}}k_{\mathrm{pol}}k_{\mathrm{pos}}\right)}_{Q_{\mathrm{clin}},Q_{\mathrm{msr}}}^{f_{\mathrm{clin}},f_{\mathrm{msr}}}\right)}.$$

where B is a parameter that depends on the dose per pulse in msr conditions. The value of B was obtained by fitting Equation (12) for IC and SS detectors using experimentally determined sets of $k_{\mathrm{ion}}$.

For ion chambers, these $k_{\mathrm{ion}}$ used for fitting were calculated by the Boag two‐voltage method^16^ (validated by Jaffe plot regressions^22^) for a modified msr field (${\mathrm{msr}}^{\prime }$) at source‐to‐surface distances (SSD) of 80, 90, 100, and 110 cm, and a constant detector depth of 10 cm. Field sizes, $f_{{\mathrm{msr}}^{\prime }}$, were determined by scaling $f_{\mathrm{msr}}$ in order to maintain a constant effective field size at the detector plane: $f_{\mathrm{msr}}^{\prime }=f_{\mathrm{msr}}\times 100/\left(\mathrm{SSD}+10\right)$. This choice was made in order: (i) to keep a unity $k_{\mathrm{stem}}$ factor ratio by minimizing changes in stem irradiation geometry, and (ii) to provide an almost equivalent phantom scatter region therefore making it possible to assume that both setups have the same effective beam quality. $k_{\mathrm{pol}}$ values were measured for all conditions and $k_{\mathrm{pos}}$ was taken as unity for $k_{\mathrm{ion}}$ determination given the large field sizes and small positioning error.

The mathematical formalism used in the TG‐51 addendum for detector‐dependent $k_{\mathrm{ion}}$ corrections of ion chambers can be applied empirically to SS detectors.^21^ The linear mathematical relationship includes a constant term and a term that depends on the absorbed dose‐per‐pulse. This type of linear behavior should be valid for SS detectors at the dose‐per‐pulse range encountered in standard RT. Therefore, $k_{\mathrm{ion}}$ from Equation (12) will also be used for correction of SS detectors' dose per pulse response dependence. For large field sizes as used for $k_{\mathrm{ion}}$ determination, the varying SSD (i.e., between 80  and 110 cm) and field size scaling will not alter beam quality at the point of measurements when going from $f_{\mathrm{msr}}$ to $f_{{\mathrm{msr}}^{\prime }}$. Thus, in that specific context, we can assume that

(13) $$k_{Q_{{\mathrm{msr}}^{\prime }},Q_{\mathrm{msr}}}^{f_{{\mathrm{msr}}^{\prime }},f_{\mathrm{msr}}}\approx 1,$$

for both SS and IC detectors. Therefore, $k_{\mathrm{ion}}$ ratios for SS detectors used for fitting B in Equation (12) were obtained from the scaled $k_{\mathrm{ion}}$ ratios of the corresponding ${\mathrm{msr}}^{\prime }$ geometry measured with an ion chamber. Using Equations (4), (9), and (10), $k_{\mathrm{ion}}$ ratios of SS detectors for msr to ${\mathrm{msr}}^{\prime }$ geometries can be approximated as

(14) $$\begin{array}{cc} {\left[{\left(k_{\mathrm{ion}}\right)}_{Q_{{\mathrm{msr}}^{\prime }},Q_{\mathrm{msr}}}^{f_{{\mathrm{msr}}^{\prime }},f_{\mathrm{msr}}}\right]}_{SS} & \approx \frac{{\left(M_{\mathrm{raw}}^{\mathrm{IC}}\right)}_{Q_{{\mathrm{msr}}^{\prime }},Q_{\mathrm{msr}}}^{f_{{\mathrm{msr}}^{\prime }},f_{\mathrm{msr}}}}{{\left(M_{\mathrm{raw}}^{\mathrm{SS}}\right)}_{Q_{{\mathrm{msr}}^{\prime }},Q_{\mathrm{msr}}}^{f_{{\mathrm{msr}}^{\prime }},f_{\mathrm{msr}}}}\\ & \;\times {\left[{\left(k_{\mathrm{ion}}k_{\mathrm{pol}}\right)}_{Q_{{\mathrm{msr}}^{\prime }},Q_{\mathrm{msr}}}^{f_{{\mathrm{msr}}^{\prime }},f_{\mathrm{msr}}}\right]}_{\mathrm{IC}}, \end{array}$$

Using these sets of $k_{\mathrm{ion}}$ ratios, B can be determined for IC and SS by inverting Equation (12) for each ${\mathrm{msr}}^{\prime }$. A single B value for each detector was then obtained by averaging the result of B for ${\mathrm{msr}}^{\prime }$ at SSDs of 80  and 110 cm. The uncertainty on B was estimated as the ratio of the maximal variation observed among all SSDs to the mean value. With B, Equation (12) was used to correct ion‐recombination effects of IC and SS detectors

### 2.3 Determination of $k_{Q_{\mathrm{clin}},Q_{\mathrm{msr}}}^{f_{\mathrm{clin}},f_{\mathrm{msr}}}$ for PRB‐0002

The field output correction factors can be derived from a chain technique of perturbation factors with respect to the dose to a point in water for a beam of quality Q and field size f, $D_{w,Q}^{f}$.^23^ For a PSD, the chain is chosen based on the work of Papaconstadopoulos et al.^24^ with the additional correction factor accounting for the ionization quenching of the scintillator, $k_{\mathrm{ioq}}$, a type II factor of influence. The perturbation chain entails four distinct geometries. The first geometry corresponds to the fully assembled PRB‐0002, including the protective jacket and clear optical fiber, in water. The second geometry is the bare sensitive volume made of plastic scintillator in water. The third geometry mimics the second, but is entirely composed of water. Finally, the last geometry represents a point in water. Equation (15) shows each step of the perturbation chain

(15) $$\begin{array}{cc} D_{w,Q}^{f} & ={\left(k_{\mathrm{vol}}\right)}_{Q}^{f}\cdot {\bar{D}}_{\mathrm{scint}(w),Q}^{f}\\ {\bar{D}}_{\mathrm{scint}(w),Q}^{f} & =\frac{{\bar{D}}_{\mathrm{scint}(w),Q}^{f}}{{\bar{D}}_{\mathrm{scint},Q}^{f}}\cdot {\bar{D}}_{\mathrm{scint},Q}^{f}={\left(P_{\mathrm{scint}}\right)}_{Q}^{f}\cdot {\bar{D}}_{\mathrm{scint},Q}^{f}\\ {\bar{D}}_{\mathrm{scint},Q}^{f} & =\frac{{\bar{D}}_{\mathrm{scint},Q}^{f}}{{\bar{D}}_{\det,Q}^{f}}\cdot {\bar{D}}_{\det,Q}^{f}={\left(P_{\mathrm{wall}}\right)}_{Q}^{f}\cdot {\bar{D}}_{\det,Q}^{f} \end{array}$$

where ${\bar{D}}_{\mathrm{scint}(w),Q}^{f}$ is the average dose in the PRB‐0002 sensitive volume geometry composed of water, ${\bar{D}}_{\mathrm{scint},Q}^{f}$ is the average dose to the PRB‐0002 sensitive volume when scintillator material composition is taken into account, and ${\bar{D}}_{\det,Q}^{f}$ is the average dose to the sensitive volume when considering the complete detector geometry. Collapsing all terms of Equation (15) leads to

(16) $$D_{w,Q}^{f}={\left(k_{\mathrm{vol}}\right)}_{Q}^{f}\cdot {\left(P_{\mathrm{scint}}\right)}_{Q}^{f}\cdot {\left(P_{\mathrm{wall}}\right)}_{Q}^{f}\cdot {\bar{D}}_{det,Q}^{f}.$$

$P_{\mathrm{wall}}$ accounts for the impact of the PRB‐0002's wall, protective jacket and optical fiber. $P_{\mathrm{scint}}$ account for the perturbations due to the variation in density and atomic composition of the scintillating material compared to water. $k_{\mathrm{vol}}$ is the volume averaging correction factor, its determination method is described in Section 2.3.2.

Using Equation (16) in Equation (8) and assuming ionization quenching is the only $k_{II}$ correction factor, the output correction factor, $k_{Q_{\mathrm{clin}},Q_{\mathrm{msr}}}^{f_{\mathrm{clin}},f_{\mathrm{msr}}}$, for PSDs takes the form

(17) $$k_{Q_{\mathrm{clin}},Q_{\mathrm{msr}}}^{f_{\mathrm{clin}},f_{\mathrm{msr}}}={\left(k_{\mathrm{vol}}P_{\mathrm{scint}}P_{\mathrm{wall}}k_{\mathrm{ioq}}\right)}_{Q_{\mathrm{clin}},Q_{\mathrm{msr}}}^{f_{\mathrm{clin}},f_{\mathrm{msr}}}$$

$P_{\mathrm{wall}}$, $P_{\mathrm{scint}}$, and $k_{\mathrm{ioq}}$ are calculated through MC simulations. $k_{\mathrm{vol}}$ is determined through experimental data as detailed in Section 2.3.2.

#### 2.3.1 Ionization quenching

In this study, ionization quenching was included as a correction factor and was calculated through MC simulations following the formalism proposed by Santurio et al.^14^ The empirical Birks law expresses the light yield per unit of path length as a function of the scintillator efficiency and the quenching parameter (kB), as follows:

(18) $$L\left(E\right)=\sum_{n=1}^{N}\left(\int_{E_{\min,n}}^{E_{\max,n}}\frac{A}{1+kB\cdot L_{\Delta }\left(E\right)}\;dE\right)$$

where N denotes the number of charged particles that interact within the PSD sensitive volume, $E_{\min,n}$ and $E_{\max,n}$ indicate the minimum and maximum energies of the *nth* particle within the sensitive volume of the detector and $L_{\Delta }\left(E\right)$ is the restricted linear electronic stopping power with an energy cutoff value Δ that was fixed at 1 keV in the MC simulations. Then the ionization quenching correction factor is the light yield ratio without and with quenching

(19) $$k_{\mathrm{ioq}}={\left[\frac{L{\left(E\right)}_{\text{ideal}}}{L{\left(E\right)}_{\text{quenching}}}\right]}_{Q_{\text{clin}},Q_{\text{msr}}}^{f_{\text{clin}},f_{\text{msr}}}.$$

In this study, kB was set to 0.019 $cmM^{-1}eV$ to estimate the effects of ionization quenching, following the results of Santurio et al.^13^ using PS‐based PSD. In addition, simulations with kB=0.01 $cmM^{-1}eV$ were also performed to evaluate the sensitivity to this parameter on $k_{\mathrm{ioq}}$.

#### 2.3.2 Positional uncertainty ($k_{\mathrm{pos}}$) and volume averaging ($k_{\mathrm{vol}}$) corrections

Lechner et al. proposed an analytical formalism to evaluate the effects of detector positional uncertainties on small field output factors.^25^ They used second‐order polynomial fit to measured dose profiles and positional uncertainty probability distribution functions to determine the expectation value of the measured dose in small fields. The approach of Lechner et al. is herein expanded to include the convolution arising from volume averaging occurring within the sensitive volume of the detector. This way, $k_{\mathrm{pos}}$ and $k_{\mathrm{vol}}$ correction factors can both be determined using the same analytical approach.

Three hypotheses are made in order to determine $k_{pos}$ and $k_{vol}$. First, the two‐dimensional relative dose in a plane perpendicular to the beam's axis in homogeneous water (i.e., in the absence of a detector) can be described by the product of two one‐dimensional second‐order polynomial functions

(20) $$D\left(x,y\right)\;=f\left(x\right)g\left(y\right)\;=\;\left(a_{0}+a_{1}x+a_{2}x^{2}\right)\;\left(b_{0}+b_{1}y+b_{2}y^{2}\right).$$

This quadratic approximation is reasonable near the central axis of the beam and has a maximum value at point $\left(x_{\max},y_{\max}\right)$. Second, the sensitive volume orientation and detector geometry are assumed to be known precisely and the measured signal is assumed to be proportional to the integral of the two‐dimensional dose distribution weighted by the height of the detector sensitive volume over a cross sectional area, A of the detector in the x−y plane

(21) $$M\left(x_{0},y_{0}\right)=\int_{A}D\left(x,y\right)h\left(x-x_{0},y-y_{0}\right)dxdy,$$

where $h(x-x_{0},y-y_{0})$ is the normalized height of the detector at position $\left(x-x_{0},y-y_{0}\right)$ from the detector's center $\left(x_{0},y_{0}\right)$. The normalization serves to get a unit measurement signal for a uniform unit dose distribution. Finally, the actual detector position is assumed to be known with an uncertainty defined by a two‐dimensional square probability density function of half‐widths $w_{x}$ and $w_{y}$. Therefore, the expectation value of a measurement signal at ($x_{0},y_{0}$) can be determined by

(22) $$\langle M(x_{0},y_{0})\rangle =\frac{1}{4w_{x}w_{y}}\int_{-w_{x}}^{w_{x}}\int_{-w_{y}}^{w_{y}}M\left(x+x_{0},y+y_{0}\right)dxdy.$$

Using these equations, the positional uncertainty and volume correction factors are

(23) $$k_{pos}=\frac{M(x_{\max},y_{\max})}{\langle M(x_{\max},y_{\max})\rangle },$$

(24) $$k_{vol}=\frac{D(x_{\max},y_{\max})}{M(x_{\max},y_{\max})},$$

The height function, h and detector cross‐sectional area, A depend on the detector sensitive volume geometry and orientation. Detector sensitive volume geometries, as defined by the manufacturers, may be either spherical, cylindrical or cylindrical with a half‐spherical tip. Orientations are either with the detector symmetry axis parallel or perpendicular to the beam axis (i.e., parallel to the z or y axis respectively). Knowing the geometry and orientation of a given detector, M and $\langle M\rangle$ can be determined analytically in terms of the $a_{i}$ and $b_{i}$ of Equation (20) as detailed in Supplementary material Section 2. These analytical equations combined with Equations (23) and (24) are used to evaluate $k_{\mathrm{pos}}$ and $k_{\mathrm{vol}}$ correction factors.

Cross‐line (x) and in‐line (y) profiles were acquired with multiple detectors and fitted simultaneously to Equation (21) to determine the $a_{i}$ and $b_{i}$ parameters. Because the two dimensional dose function of Equation (20) is independent of detector geometry and orientations, the fitted parameters obtained by fitting experimental data from one detector can be used to evaluate correction factors for any other detector. Similarly to the work by Lechner et al.,^25^ the variance of $M(x_{0},y_{0})$ can also be analytically obtained from an integral equation similar to Equation (22) but for: $\langle M^{2}\rangle -{\langle M\rangle }^{2}$. This variance has been used to evaluate the statistical distribution uncertainty on $k_{\mathrm{pos}}$.

#### 2.3.3 Monte Carlo simulations

MC calculations of absorbed dose‐to‐detector were performed with the user code egs_chamber ^26^ from EGSnrc.^27^, ^28^ The phase space of the Varian Truebeam 6 MV photon beam above the jaws was provided by the manufacturer. To generate phase spaces at specific field size, the jaws were simulated according to the manufacturer specifications with BEAMnrc.^29^ New phase spaces at specific field sizes were scored at 75 cm from the source. These phase spaces were validated by comparing simulated profiles and output factors to published data^14^, ^30^ and experimental profiles.

The PRB‐0002 described in Section 2.4.1 was simulated based on data provided by the manufacturer. It was placed inside a water phantom (60 × 60 × 40 $\;cm^{3}$) at 10 cm depth. The SSD was 90 cm, and field sizes were 30, 10, 8, 6, 5, 4, 2, 1, 0.8, and 0.6 cm. MC parameters are presented in Table 1, along with the variance reduction techniques used to reduce calculation time. The number of histories is such that the statistical uncertainty of each MC simulation is between 0.1% and 0.2%.

**TABLE 1:** Record of Monte Carlo simulations characteristics and parameters used in EGSnrc.

Item	EGSnrc
MC code	egs_chamber, EGSnrc 2021 release
Validation	Previously validated by Wulff et al.^26^
Scored quantities	Dose, light yield and cummulative dose
Source description	Phase space of a Varian Truebeam 6 MV photon beam
Cross sections	Default
Transport parameters	Photon cutoff 0.001 MeV
	Electron cutoff 0.512 MeV
Variance reduction techniques	Photon cross‐section enhancement
	Intermediate phase‐space storage
	Range rejection and Russian Roulette
	ESAVE=ECUT=0.512 MeV
Statistical uncertainty	0.1%–0.2% in the sensitive volume
Statistical methods	Standard deviation of independent parallel simulations

For ionization quenching calculation, two Ausgab objects (AO) were implemented to compute the light yield and the cumulative dose spectrum. These AO were constructed in compliance with the guidelines presented in reference^14^ and corroborated through direct communication with the author.

### 2.4 Detectors

#### 2.4.1 Plastic scintillation detector system

The Hyperscint RP‐200 is a multi‐channel scintillation dosimetry platform from Medscint Inc. One channel was used in this study to read a PRB‐0002 which is composed of a proprietary plastic scintillator coupled to a 20 m clear plastic optical fiber guiding the optical signal outside the treatment room to the spectral optical reader. The sensitive volume has a cylindrical shape of 1 mm of diameter by 1 mm length. The detector calibration was performed as described by the manufacturer. It includes measurements of the individual spectral components from the scintillation, Cherenkov, fluorescence and spectral attenuation of the optical fiber in order to allow for a correct stem removal. To do so, the linac kV source was used to separate scintillation and fluorescence spectra. Then, the MV beam is used to stimulate the emission of Cherenkov, scintillation and fluorescence simultaneously. This is repeated at two positions on the optical fiber to account for spectral attenuation of Cherenkov light. Finally, the MV beam is also used with a 10×10 ${\mathrm{cm}}^{2}$ field at $d_{\max}$ to obtain a normalization factor for the scintillation signal (i.e., the amount of scintillation per unit dose) which is proportional to dose. All PRB‐0002 dose measurements were acquired at a frame rate of 1 Hz. Seven probes, each with a 1 mm diameter and of the same model, were used paired with either of the two available readers to assess inter‐detector variations.

#### 2.4.2 IC and SS detectors

Detector‐specific field output correction factors have been extracted for several detectors of different sizes and properties, with dimensions and characteristics well‐suited for small field dosimetry, as recommended in the TRS‐483^4^ report. The following micro‐ICs were used: two IBA Razor Nano chambers (IBA RAZNC), two IBA Razor chambers (IBA RAZC) and two Standard Imaging Exradin A26 (SI A26) chambers. Furthermore, two synthetic micro diamond detectors from PTW, model 60019 (PTW 60019), and three unshielded IBA Razor diodes (IBA RAZD) were also used. Relevant detector physical properties are listed in Table 2. Charges were collected with a Supermax^TM^ electrometer (Standard Imaging, Middleton, WI, USA) for all detectors except for the PRB‐0002 that used a dedicated spectral reader. ICs were operated at ±300 V.

**TABLE 2:** Physical properties of IC and SS detectors studied for detector‐specific field output correction factor.

			Active volume	Sensitive	Wall material	Central electrode
Name	Type	Active volume	length/radius (mm)	material (g/${\mathrm{cm}}^{3}$)	(g/${\mathrm{cm}}^{3}$)	material (g/${\mathrm{cm}}^{3}$)
IBA RAZNC	Ion chamber	Spherical, 3 ${\mathrm{mm}}^{3}$	− / 1.0	Air (0.001)	C552 (1.76)	Graphite (2.26)
IBA RAZC	Ion chamber	Cyl/half‐sph, 11 ${\mathrm{mm}}^{3}$	2.6 / 1.0	Air (0.001)	C552 (1.76)	Graphite (2.26)
SI A26	Ion chamber	Spherical, 15 ${\mathrm{mm}}^{3}$	− / 1.65	Air (0.001)	C552 (1.76)	C552 (1.76)
IBA RAZD	Unshielded diode	Cylindrical, 0.006 ${\mathrm{mm}}^{3}$	0.02 / 0.3	p‐Type silicon (2.33)	ABS (1.05)	−
PTW 60019	Synthetic diamond	Cylindrical, 0.004 ${\mathrm{mm}}^{3}$	0.001 / 1.1	Diamond (3.52)	RW3 (1.05)	−
PRB‐0002	PSD	Cylindrical, 0.79 ${\mathrm{mm}}^{3}$	1 / 0.5	Polystyrene with dopants (1.06)	Nylon (1.01)	−

For ICs, measurements at both +300 V and ‐300 V bias were performed for each field size in order to determine $k_{\mathrm{pol}}$. The ion recombination correction was applied as described in Section 2.2 to all detectors using a B parameter determined by fitting $k_{\mathrm{ion}}$ values obtained from the Boag two‐voltage (150 V/300 V) method of a Standard Imaging A26 IC.

### 2.5 Measurement methodologies

#### 2.5.1 Experimental setup

All measurements were performed on a Varian Truebeam linac (Varian Medical Systems, Palo Alto, CA, USA) using a 6 MV flattened photon beam. Output factor measurements were performed by placing the effective point of measurement of a given detector at the linac isocenter, at 10 cm water depth in a motorized IBA Smartscan water tank (IBA Dosimetry, Schwarzenbruck, Germany). The SSD was set to 90 cm and was adjusted using the magnetic front pointer with the gantry placed upright at 0°. Each detector's symmetry axis was aligned parallel to the beam axis, with the stem at deeper depths for all detectors except PSDs. For PSD, the optical fiber was aligned perpendicular to the beam axis (in‐line direction), in order to minimize stem signal.

Centering of detectors was performed with the “11‐points” methodology, described in Section 2.5.2. Measurements were performed for twelve jaw‐delimited square fields with side lengths of 0.6, 0.8, 1.0, 1.5, 2.0, 3.0, 4.0, 5.0, 6.0, 8.0, 10.0, and 30.0 cm. One hundred Monitor units (MU) were delivered for each irradiation and at least 3 irradiations were performed for each field size. The 10 × 10 ${\mathrm{cm}}^{2}$ field size was considered the reference field size (msr=ref=10×10 ${\mathrm{cm}}^{2}$) and has been measured frequently in order to be used as a normalization field and to reduce the impact of machine output, temperature and pressure drifts.

#### 2.5.2 Equivalent square field size and detector centering method

For each nominal field size, actual measured field sizes, ${\mathrm{FS}}_{x}$ and ${\mathrm{FS}}_{y}$, were converted to the equivalent measured square small field size $S_{\mathrm{clin}}$ following the approach adopted by TRS‐483^4^ where $S_{\mathrm{clin}}$ is given by:

(25) $$S_{\text{clin}}=\sqrt{{\mathrm{FS}}_{x}\times {\mathrm{FS}}_{y}},$$

where ${\mathrm{FS}}_{x}$ corresponds to the radiation field full width at half maximum (FWHM) in cross‐line direction, x, and ${\mathrm{FS}}_{y}$ (FWHM) for in‐line direction, y, perpendicular to the former. ${\mathrm{FS}}_{x}$ and ${\mathrm{FS}}_{y}$ were determined for all field sizes using an 11‐points technique allowing both equivalent field size measurement and precise detector centering. First, the detector is visually centered. Then, for cross‐line orientation, in a step‐and‐shoot operation, two measurements of 100 MU are acquired on each side of the field at positions where the dose is estimated to be close to 30% and 70% of the profile's maximum dose. Assuming linear profile slopes in these penumbra regions, the cross‐line field center position is determined. The detector is moved to that position and then a fifth measurement is acquired for cross‐line profile values normalization and FWHM (${\mathrm{FS}}_{x}$) calculation. The procedure is repeated for in‐line orientation, adding five other measurements. Finally, the 11^th^ measurement is acquired at the precise field center which is now known in both directions.

#### 2.5.3 Analytical functions of $M_{Q_{\mathrm{clin}},Q_{\mathrm{ref}}}^{f_{\mathrm{clin}},f_{\mathrm{ref}}}$ and $k_{Q_{\mathrm{clin}},Q_{\mathrm{msr}}}^{f_{\mathrm{clin}},f_{\mathrm{msr}}}$

In order to be able to calculate field output correction factor for any given $S_{\mathrm{clin}}$, values from evaluated $M_{Q_{\mathrm{clin}},Q_{\mathrm{ref}}}^{f_{\mathrm{clin}},f_{\mathrm{ref}}}$ and $k_{Q_{\mathrm{clin}},Q_{\mathrm{msr}}}^{f_{\mathrm{clin}},f_{\mathrm{msr}}}$ at measured $S_{\mathrm{clin}}$ were fitted with analytical functions optimized with a bounded non‐linear least square trust region reflective algorithm.^31^, ^32^ First, $M_{Q_{\mathrm{clin}},Q_{\mathrm{ref}}}^{f_{\mathrm{clin}},f_{\mathrm{ref}}}$ is fitted with the function given in Sauer and Wilbert^33^

(26) $$M_{Q_{\mathrm{clin}},Q_{\mathrm{ref}}}^{f_{\mathrm{clin}},f_{\mathrm{ref}}}\left(S_{\text{clin}\;}\right)=P_{\infty }\frac{S_{\text{clin}\;}^{n}}{l^{n}+S_{clin}^{n}}+S_{\infty }\left(1-e^{-b\cdot S_{\mathrm{clin}}}\right),$$

where l, n, b, $P_{\infty }$, $S_{\infty }$ are fitting parameters. Equation (26) is normalized so that $M_{Q_{\mathrm{clin}},Q_{\mathrm{ref}}}^{f_{\mathrm{clin}},f_{\mathrm{ref}}}$(10 cm) is unity. $k_{Q_{\mathrm{clin}},Q_{\mathrm{msr}}}^{f_{\mathrm{clin}},f_{\mathrm{msr}}}$ is then fitted with the function given in the TRS‐483 report^4^

(27) $$k_{Q_{\mathrm{clin}},Q_{\mathrm{msr}}}^{f_{\mathrm{clin}},f_{\mathrm{msr}}}\left(S_{\mathrm{clin}}\right)=\frac{1+a_{4}e^{-\frac{10-a_{1}}{a_{2}}}}{1+a_{4}e^{-\frac{S_{\mathrm{clin}}-a_{1}}{a_{2}}}}+a_{3}\left(S_{\mathrm{clin}}-10\right),$$

where $a_{1}$, $a_{2}$, $a_{3}$ and, $a_{4}$ are fitting parameters.

### 2.6 Uncertainties

A careful uncertainty analysis is necessary to quantitatively evaluate the field output correction factors obtained for PRB‐0002 and compare it to other detectors. Uncertainty analysis was performed for (1) the reference PRB‐0002 $k_{Q_{\mathrm{clin}},Q_{\mathrm{msr}}}^{f_{\mathrm{clin}},f_{\mathrm{msr}}}$ (2) the $k_{Q_{\mathrm{clin}},Q_{\mathrm{msr}}}^{f_{\mathrm{clin}},f_{\mathrm{msr}}}$ derived with other detectors and (3) the measured field output factor.

#### 2.6.1 PRB‐0002 uncertainty on $k_{Q_{\mathrm{clin}},Q_{\mathrm{msr}}}^{f_{\mathrm{clin}},f_{\mathrm{msr}}}$

Field output correction factors for PRB‐0002 are determined with Equation (17). Three parameters come from MC simulations: $P_{wall}$, $P_{\mathrm{scint}}$, and $k_{\mathrm{ioq}}$ ratios. Type A statistical uncertainties (i.e., uncertainties derived using statistical analysis of a series of observations) on these three ratios are approximately 0.1%, 0.1%, and 0.35% respectively. Two sources of type B uncertainties were also considered on these ratios. The first arises from inaccuracies on physical data used in cross‐sections. Studies have investigated the impact of systematic uncertainty from physical data on the beam quality correction factor $k_{Q,Q_{0}}$ for ICs.^34^, ^35^ Wulff et al.^35^ suggested a systematic uncertainty of 0.2% for 6 MV photon beams. However, this value is dominated by the uncertainty on graphite I‐value. Despite graphite being absent from PSDs, this systematic uncertainty was used in this study. Furthermore, changes in beam quality resulting from changes in field sizes (i.e., changes between $Q_{\mathrm{msr}}$ and $Q_{\mathrm{clin}}$) are expected to be smaller than changes between $Q_{0}$ and Q that were the focus of the work of Wulff et al. Thus, this 0.2% type B uncertainty for cross sections inaccuracies is a conservative estimate. The second source of type B uncertainty arises from geometric and density differences between the actual detector and the MC model. This uncertainty was determined, as suggested by others,^34^, ^36^ by performing simulations with sensitive volume with diameters varying by ±5%.

The last factor affecting $k_{Q_{\mathrm{clin}},Q_{\mathrm{msr}}}^{f_{\mathrm{clin}},f_{\mathrm{msr}}}$ uncertainty is the experimental ${\left(k_{\mathrm{vol}}\right)}_{Q_{\mathrm{clin}},Q_{\mathrm{msr}}}^{f_{\mathrm{clin}},f_{\mathrm{msr}}}$ ratio. Uncertainties on this factor come from two sources: (1) type A statistical component evaluated with the standard deviation of the mean of Equation (24) from several detectors profile measurements, and (2) type B component that is due to geometric uncertainties, and is evaluated, as previously, with a ±5% geometric parameters variation. The total combined uncertainty on reference PRB‐0002 field output correction factor is evaluated by summing in quadrature all those contributions.

#### 2.6.2 Other detectors uncertainty on derived $k_{Q_{\mathrm{clin}},Q_{\mathrm{msr}}}^{f_{\mathrm{clin}},f_{\mathrm{msr}}}$

Any other detector field output correction factor derived from comparative measurements with Medscint's PRB‐0002, using Equation (4) depends on five factors for which the uncertainties must be evaluated. The first factor is the PRB‐0002's $k_{Q_{\mathrm{clin}},Q_{\mathrm{msr}}}^{f_{\mathrm{clin}},f_{\mathrm{msr}}}$ (see Section 2.6.1). The four other factors are for the corrected measurements. The relative uncertainty of each parameter required for measurement correction (i.e., Equation 9 for IC, Equation 10 for SS, and Equation 11 for PSD) expressed as a ratio between clin and msr fields must be considered.

Type A statistical uncertainty of $M_{raw}$ ratios (or $k_{\mathrm{pol}}M_{raw}$ ratios for IC detectors) was taken as the standard deviation of the mean from multiple measurements taken with several detectors of the same model. Those variations in measured raw values are expected to come mainly from readout and linac dose short term reproducibility as well as detector and jaws positioning reproducibility. Combined type B uncertainty for temperature, pressure, humidity, readout, background and machine drift correction ratios have been estimated to be at most 0.1%. Type B uncertainty on $k_{\mathrm{ion}}$ correction ratio has been estimated using a worst case scenario from Equation (12) with B ranging from lowest to highest estimated values between all tested SSDs. Type A and B uncertainties in $k_{\mathrm{pos}}$ ratio were evaluated similarly as those on $k_{vol}$ ratio described in Section 2.6.1.

For $k_{\mathrm{stem}}$ of PRB‐0002 measurements, a sensitivity analysis estimated the overall uncertainty. Using the experimental noise of each wavelength of each spectrum acquired during the calibration procedure, two hundred different calibration sets were generated. Each set was applied to correct the stem for each field size measurement. The relative standard deviation of the distribution at larger field sizes was found to be lower than 0.1%. For other types of detector, the $k_{\mathrm{stem}}$ uncertainty is estimated to be lower than 0.1%. Furthermore, the vertical orientation was used to minimize $k_{\mathrm{stem}}$ deviation between field sizes. Conservatively, a value of 0.1% was used.

Finally, because the derived field output correction factors are associated, tabulated and fitted with an effective square field size dependence, the uncertainty on the field size determination must be propagated to the $k_{Q_{\mathrm{clin}},Q_{\mathrm{msr}}}^{f_{\mathrm{clin}},f_{\mathrm{msr}}}$ uncertainty. Therefore, the derivative of the fitted corrected measurement ratio with field size is calculated and multiplied by the field size determination uncertainty to obtain that component of $k_{Q_{\mathrm{clin}},Q_{\mathrm{msr}}}^{f_{\mathrm{clin}},f_{\mathrm{msr}}}$ total uncertainty. The equivalent square field size determination uncertainty has been evaluated by taking the standard deviation of a series of five consecutive ${\mathrm{FS}}_{x}$ and ${\mathrm{FS}}_{y}$ measurements using the method described in Section 2.5.2 for a fixed 0.6 × 0.6 ${\mathrm{cm}}^{2}$ nominal field. The effective square field size uncertainty is obtained by combining uncertainties on ${\mathrm{FS}}_{x}$ and ${\mathrm{FS}}_{y}$ using the derivative of Equation (25).

Estimation of the detector central position uncertainty is done by using the standard deviation of the calculated central position from the previously described series of five consecutive field size measurements with the same fixed nominal field. This position uncertainty must further be combined with the inherent positioning system uncertainty that will ultimately affect the final detector effective position. The total positioning uncertainty is assumed here to have a square probability distribution with half‐width $w_{x}$ and $w_{y}$.

The uncertainty on the corrected measurement ratio due to detector positioning is estimated with the standard deviation of the mean of $k_{\mathrm{pos}}$ (evaluated from $w_{x}$ and $w_{y}$ values) for the number of times a detector has been positioned for that field. To estimate the uncertainty on the corrected measurement ratio due to jaws positioning when a detector is centered after each jaw movement, the field size uncertainty due to jaws repositioning is first evaluated then multiplied by the derivative of the corrected measurement ratio at the measured field size. The field size uncertainty due to jaw repositioning is evaluated with a series of five consecutive field size measurements with the same 0.6 × 0.6 ${\mathrm{cm}}^{2}$ nominal field but with jaw movements between each field size measurement.

#### 2.6.3 Field output factor uncertainty

The last part of the uncertainty analysis consists of evaluating the measured field output factor uncertainty. Following Equation (2), three terms are involved. The first one is the uncertainty on the detector $k_{Q_{\mathrm{clin}},Q_{\mathrm{msr}}}^{f_{\mathrm{clin}},f_{\mathrm{msr}}}$ ratio (see Sections 2.6.1 and 2.6.2). The two other factors are the uncertainties on clin and msr corrected measurements. The combined uncertainty of the corrected measurement ratio is determined as described in previous sections. The total combined uncertainty on field output factor measurements is evaluated by summing in quadrature the three terms' contribution.

In the case where $k_{Q_{\mathrm{clin}},Q_{\mathrm{msr}}}^{f_{\mathrm{clin}},f_{\mathrm{msr}}}$ correction factor is extracted from the fitted function in Equation (27), the uncertainty on the extracted value can be evaluated with a MC statistical sampling method. The curve fitting procedure was repeated a thousand times with $k_{Q_{\mathrm{clin}},Q_{\mathrm{msr}}}^{f_{\mathrm{clin}},f_{\mathrm{msr}}}$ values sampled from a gaussian distributions with measured $k_{Q_{\mathrm{clin}},Q_{\mathrm{msr}}}^{f_{\mathrm{clin}},f_{\mathrm{msr}}}$ as mean with a standard deviation equal to the uncertainty calculated from one of the last two sections. The estimated uncertainty is divided in two parts; the first involves all components of random nature potentially affecting differently each $k_{Q_{\mathrm{clin}},Q_{\mathrm{msr}}}^{f_{\mathrm{clin}},f_{\mathrm{msr}}}\left(S_{\mathrm{clin}}\right)$ and the second involves the remaining uncertainty components that could affect all $k_{Q_{\mathrm{clin}},Q_{\mathrm{msr}}}^{f_{\mathrm{clin}},f_{\mathrm{msr}}}\left(S_{\mathrm{clin}}\right)$ values systematically. Therefore, the resampling of $k_{Q_{\mathrm{clin}},Q_{\mathrm{msr}}}^{f_{\mathrm{clin}},f_{\mathrm{msr}}}$ values at each iteration is performed in two steps. A first random number is used to sample the relative deviation affecting values at all $S_{\mathrm{clin}}$, then new random numbers are drawn for each $S_{\mathrm{clin}}$ and are used to sample the random portion of the estimated uncertainties. The two values are added to obtain the resampled $k_{Q_{\mathrm{clin}},Q_{\mathrm{msr}}}^{f_{\mathrm{clin}},f_{\mathrm{msr}}}\left(S_{\mathrm{clin}}\right)$ values for each iteration. Standard deviation of the resulting fits at every 0.5 mm for $S_{\mathrm{clin}}$ between 0.5  and 40.0 cm were determined and then used as $k_{Q_{\mathrm{clin}},Q_{\mathrm{msr}}}^{f_{\mathrm{clin}},f_{\mathrm{msr}}}$ fit uncertainties.

### 2.7 $k_{Q_{\mathrm{clin}},Q_{\mathrm{msr}}}^{f_{\mathrm{clin}},f_{\mathrm{msr}}}$ determination for all detectors

This section describes the process used to determine $k_{Q_{\mathrm{clin}},Q_{\mathrm{msr}}}^{f_{\mathrm{clin}},f_{\mathrm{msr}}}$ correction factors. This is first done for the detector used as a reference (PRB‐0002) as detailed in Section 2.3 and then for all other detectors.

Following this and using Equation (17), $k_{Q_{\mathrm{clin}},Q_{\mathrm{msr}}}^{f_{\mathrm{clin}},f_{\mathrm{msr}}}$ values and the associated uncertainties of the reference (PRB‐0002) can be determined for the selected set of field sizes. These values are then fitted to a second degree polynomial to interpolate them at any field size. Once $k_{Q_{\mathrm{clin}},Q_{\mathrm{msr}}}^{f_{\mathrm{clin}},f_{\mathrm{msr}}}$ is known for the reference, the other detectors, readout equipment and positioning system are characterized. For each detector (including the reference PRB‐0002), output factor measurements are acquired for all field sizes. Each measurement is preceded by field size and field center position determination (see Section 2.5.2). From these, field output correction factors and their uncertainties can be determined using Equation (4). Finally, $k_{Q_{\mathrm{clin}},Q_{\mathrm{msr}}}^{f_{\mathrm{clin}},f_{\mathrm{msr}}}$ data is fitted using Equation (27) and the associated fit uncertainties can be determined with the sampling method described in Section 2.6.3. Comparison with literature data can then be performed at specific field sizes, using the fit data and two‐sided unpaired Welch's unequal variances t‐test.^31^, ^37^

## 3 RESULTS

### 3.1 Measurement corrections

**FIGURE 1:**
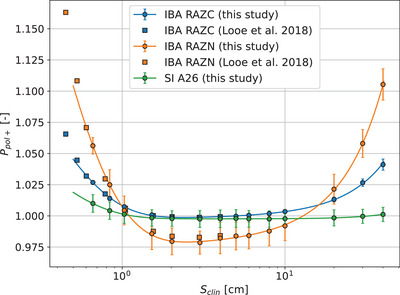
Comparison of polarity correction factor, $k_{\mathrm{pol}}$, for the ionization chambers: IBA Razor, IBA Razor Nano and Standard Imaging Exradin A26. The fit models $k_{\mathrm{pol}}$ behavior with a modified exponential base, complemented by linear and quadratic terms. Results extracted from Looe et al.^38^ are also added to the figure but reversed according to the different polarity sign convention used.

The choice of a parallel detector orientation is based on the need to keep $k_{\mathrm{stem}}$ close to unity. However this orientation also causes larger polarity corrections with varying field size. This choice of orientation nevertheless allowed for more precise measurements because the established polarity correction technique is efficient and accurate as opposed to a precise evaluation of $k_{\mathrm{stem}}$ which can be tricky. The polarity relationship for a range of field sizes is depicted in Figure 1 for three detectors: IBA Razor Chamber, IBA Razor Nano Chamber, and Standard Imaging Exradin A26. Results for IBA chambers are in good agreement with those from Looe et al.^38^ Among studied detectors, the IBA Razor Nano's $k_{\mathrm{pol}}$ exhibits the largest variation with field size with values increasing beyond 10% at both small and large fields. The SI Exradin A26 had $k_{\mathrm{pol}}$ values closer to unity with deviations mainly observed at the smallest fields.

Table 3 presents the fitted parameter B from Equation (12) as well as calculated $k_{\mathrm{ion}}$ ratios of three field sizes for IC and SS detectors.

**TABLE 3:** Values for parameter B determined using Equation (12) and ${\left(k_{\mathrm{ion}}\right)}_{Q_{\mathrm{clin}},Q_{\mathrm{msr}}}^{f_{\mathrm{clin}},f_{\mathrm{msr}}}$ for each detector at 0.6,1.0, and 2.0 cm side of squared field sizes.

Detector	B	${\left(k_{\mathrm{ion}}\right)}_{Q_{\mathrm{clin}},Q_{\mathrm{msr}}}^{f_{\mathrm{clin}},f_{\mathrm{msr}}}$		
		(0.6 × 0.6 $\;cm^{2}$)	(1 × 1 $\;cm^{2}$)	(2 × 2 $\;cm^{2}$)
IBA RAZNC	0.0035 (7)	0.9984 (3)	0.9990 (2)	0.9993 (1)
IBA RAZC	−0.001 (1)	1.0004 (4)	1.0003 (3)	1.0002 (2)
SI A26	0.0004 (1)	0.9998 (1)	0.9999 (1)	0.9999 (1)
IBA RAZD	−0.005 (1)	1.0022 (1)	1.0016 (1)	1.0011 (1)
PTW 60019	−0.003 (2)	1.0011 (5)	1.0008 (3)	1.0005 (2)

*Note*: Uncertainties are shown in brackets and represent absolute uncertainties in the last digit.

Calculations for $k_{\mathrm{pos}}$ for all detectors geometries and orientations were found to be 1.000 with negligible uncertainty. Furthermore, for Medscint's PRB‐0002, the sensitivity study testing the impact of ±5% variation in the sensitive volume radius on $k_{\mathrm{pos}}$ did not reveal any change in $k_{\mathrm{pos}}$. Therefore, a $k_{\mathrm{pos}}$ value of 1.000 was used throughout this work.

#### 3.1.1 Output factors corrected for type I influence factors

**FIGURE 2:**
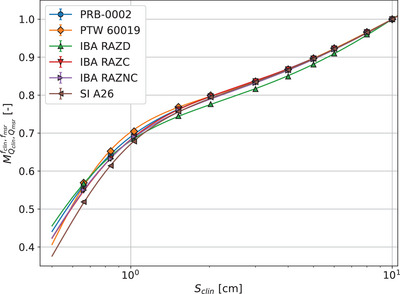
Measurement ratios corrected only for type I factors, $M_{Q_{\mathrm{clin}},Q_{\mathrm{ref}}}^{f_{\mathrm{clin}},f_{\mathrm{ref}}}$. The regression combines a sigmoid saturation model and an exponential decay component.

Measurements ratio for all detectors corrected by influence factors that are not affected by change in beam quality (i.e., type I factors) as detailed in Equations (9)–(11), $M_{Q_{\mathrm{clin}},Q_{\mathrm{ref}}}^{f_{\mathrm{clin}},f_{\mathrm{ref}}}$, is presented in Figure 2. The fit was performed using Equation (26). The corrected measurement ratios of the IBA Razor diode distinctly deviates from the trend of the other detectors. This is possibly due to an over‐response to lower energy components present for fields larger than 5 × 5 $\;cm^{2}$. As field size decreases, there is a pronounced dispersion in the $M_{Q_{\mathrm{clin}},Q_{\mathrm{ref}}}^{f_{\mathrm{clin}},f_{\mathrm{ref}}}$ values across detectors, with the SI A26 exhibiting the most significant variation.

### 3.2 Determination of $k_{Q_{\mathrm{clin}},Q_{\mathrm{msr}}}^{f_{\mathrm{clin}},f_{\mathrm{msr}}}$ for PRB‐0002

**FIGURE 3:**
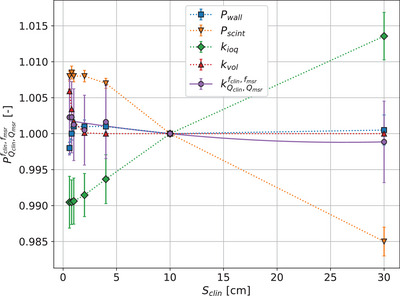
Monte Carlo calculated perturbation and correction factors of the PRB‐0002, including $k_{Q_{\mathrm{clin}},Q_{\mathrm{msr}}}^{f_{\mathrm{clin}},f_{\mathrm{msr}}}$. $P_{\mathrm{wall}}$ accounts for the perturbations coming from the extracameral components, $P_{\mathrm{scint}}$ for the perturbations due to variation in density and atomic composition. $k_{\mathrm{ioq}}$ is the ionization quenching correction factor and $k_{\mathrm{vol}}$ is the volume averaging correction factor determined by dose profiles measurements. The machine specific reference, msr, field is $10\times 10\;cm^{2}$.

Medscint's PRB‐0002 output correction factors, $k_{Q_{\mathrm{clin}},Q_{\mathrm{msr}}}^{f_{\mathrm{clin}},f_{\mathrm{msr}}}$ obtained from Equation (17), along with the correction and perturbation factors ($k_{\mathrm{ioq}}$, $k_{\mathrm{vol}}$, $P_{\mathrm{wall}}$, $P_{\mathrm{scint}}$), are shown in Figure 3 for field sizes ranging from 0.6 × 0.6 to 30 × 30 $\;cm^{2}$. It can be seen that $P_{\mathrm{scint}}$ and $k_{\mathrm{ioq}}$ offset each other, resulting in PRB‐0002's $k_{Q_{\mathrm{clin}},Q_{\mathrm{msr}}}^{f_{\mathrm{clin}},f_{\mathrm{msr}}}$ being primarily influenced by $k_{\mathrm{vol}}$. The uncertainties for $P_{\mathrm{wall}}$ and $P_{\mathrm{scint}}$ are both 0.1% and uncertainty for $k_{\mathrm{ioq}}$ is 0.36%, leading to a total uncertainty of 0.4% on $k_{Q_{\mathrm{clin}},Q_{\mathrm{msr}}}^{f_{\mathrm{clin}},f_{\mathrm{msr}}}$. The sensitivity of $k_{\mathrm{ioq}}$ to variations in kB (see Section 2.3.1) showed no statistical difference.

$k_{\mathrm{vol}}$ is derived using Equation (24) in conjunction with dose profiles measured by all detectors oriented parallel to the beam axis (see Supplementary material Section 3). The impact of each detector on the measured profile was minimized by applying the correction described in Section 2.3.2 and Supplementary material Section 2. The value of $k_{\mathrm{vol}}$ for PRB‐0002 was taken as the average over all detectors.

### 3.3 Uncertainties

Uncertainty budget for $k_{Q_{\mathrm{clin}},Q_{\mathrm{msr}}}^{f_{\mathrm{clin}},f_{\mathrm{msr}}}$ and $\Omega_{Q_{\mathrm{clin}},Q_{\mathrm{msr}}}^{f_{\mathrm{clin}},f_{\mathrm{msr}}}$ measurements with the PRB‐0002 is detailed in Tables 4 and 5. Equivalently, uncertainty budget of the PTW 60019 microDiamond detector is shown in Tables 6 and 7 as another example. These budgets enumerate the sources of uncertainty that are expected to contribute to the combined uncertainty, presented with a one‐sigma probability coverage (*k* = 1), for three selected field sizes.

**TABLE 4:** Example uncertainty budget for determining $k_{Q_{\mathrm{clin}},Q_{\mathrm{msr}}}^{f_{\mathrm{clin}},f_{\mathrm{msr}}}$ with the PRB‐0002.

Item	Type	Field size [${\mathrm{cm}}^{2}$]		
		0.6 × 0.6	1 × 1	2 × 2
Monte Carlo				
Statistical $P_{\mathrm{wall}}$	A	0.08%	0.09%	0.09%
Statistical $P_{\mathrm{scint}}$	A	0.07%	0.08%	0.08%
Statistical $k_{\mathrm{ioq}}$	A	0.36%	0.32%	0.30%
Cross‐section^35^	B	0.2%	0.2%	0.2%
Geometry and Density^36^	B	0.3%	0.3%	0.3%
Experimental/Analytical				
Statistical $k_{\mathrm{vol}}$ (7 dets)	A	0.026%	0.009%	0.002%
Geometry $k_{\mathrm{vol}}$	B	0.03%	0.005%	0.001%
Combined uncertainty (k=1)		0.52%	0.50%	0.48%

*Note*: This includes statistical uncertainties for each factor from Monte Carlo simulations, overall uncertainty due to physical data (e.g., cross‐section), and uncertainties between actual and modeled density and geometry. For the hybrid method of calculating $k_{\mathrm{vol}}$, uncertainties include statistical variation from measurements and geometrical differences between actual and theoretical geometries.

**TABLE 5:** Uncertainty budget example for $\Omega_{Q_{\mathrm{clin}},Q_{\mathrm{msr}}}^{f_{\mathrm{clin}},f_{\mathrm{msr}}}$ measurements with the PRB‐0002.

Item	Type	Field size [${\mathrm{cm}}^{2}$]		
		0.6 × 0.6	1 × 1	2 × 2
$k_{Q_{\mathrm{clin}},Q_{\mathrm{msr}}}^{f_{\mathrm{clin}},f_{\mathrm{msr}}}$ PSD (see Table 4) □★	B	0.52%	0.50%	0.48%
$M_{f_{\mathrm{clin}},f_{\mathrm{msr}}}$				
Statistical $M_{raw}$ (1 meas) □	A	0.29%	0.16%	0.11%
Statistical $M_{raw}$ (7 dets × 2 meas) ★	A	0.08%	0.04%	0.03%
$k_{stem}\;□\;★$	B	0.1%	0.1%	0.1%
$k_{other}\;□\;★$	B	0.1%	0.1%	0.1%
Statistical $k_{pos}$ (7 dets) □★	A	−	−	−
Geometry $k_{pos}\;□\;★$	B	−	−	−
${\left(M_{raw}\right)}_{f_{\mathrm{clin}},f_{\mathrm{msr}}}$ (single det.)				
Positioning system/FS center $w_{x}$/$w_{y}$	B		0.1/0.1 mm	
Positioning system/FS center	B	0.02%	0.01%	−
Readout/Machine reproducibility	A	0.1%	0.1%	0.1%
Jaws reproducibility ${\mathrm{FS}}_{x}$/${\mathrm{FS}}_{y}$	A		0.02/0.05 mm	
Jaws reproducibility $S_{\mathrm{clin}}$	A	0.026 mm (0.28%)	0.027 mm (0.08%)	0.027 mm (0.02%)
Combined uncertainty (k=1)		0.30%	0.13%	0.10%
Field size determination				
Statistical ${\mathrm{FS}}_{x}$/${\mathrm{FS}}_{y}$ (1 centering)	A		0.06/0.07 mm	
Statistical ${\mathrm{FS}}_{x}$/${\mathrm{FS}}_{y}$ (7 centerings)	A		0.02/0.03 mm	
Inter‐det‐type ${\mathrm{FS}}_{x}$/${\mathrm{FS}}_{y}$	B		0.07/0.08 mm	
$S_{\mathrm{clin}}$ (1 centering) □	A	0.071 mm (0.75%)	0.071 mm (0.22%)	0.071 mm (0.05%)
$S_{\mathrm{clin}}$ (7 centerings) ★	A	0.053 mm (0.57%)	0.053 mm (0.16%)	0.053 mm (0.04%)
Combined uncertainty (k=1) □		0.97%	0.59%	0.51%
Combined uncertainty (k=1) ★		0.79%	0.55%	0.50%

*Note*: Sources of uncertainty of $M_{f_{\mathrm{clin}},f_{\mathrm{msr}}}$ ${\left(M_{\mathrm{raw}}\right)}_{f_{\mathrm{clin}},f_{\mathrm{msr}}}$ for a single detector and field determination are explicitly detailed. The ★ symbol denotes uncertainties from seven detectors with two measurements each, and the □ symbol indicates uncertainty from a single measurement with one detector.

**TABLE 6:** Uncertainty budget example for $k_{Q_{\mathrm{clin}},Q_{\mathrm{msr}}}^{f_{\mathrm{clin}},f_{\mathrm{msr}}}$ determination of the PTW60019 detector.

Item	Type	Field size [${\mathrm{cm}}^{2}$]		
		0.6 × 0.6	1 × 1	2 × 2
$\Omega_{Q_{\mathrm{clin}},Q_{\mathrm{msr}}}^{f_{\mathrm{clin}},f_{\mathrm{msr}}}$ PSD (see Table 5) □★	B	0.79%	0.55%	0.50%
$M_{f_{\mathrm{clin}},f_{\mathrm{msr}}}$ PTW 60019				
Statistical $M_{\mathrm{raw}}$ (1 meas) □	A	1.3%	0.42%	0.10%
Statistical $M_{\mathrm{raw}}$ (2 dets × 4 meas) ★	A	0.46%	0.15%	0.04%
$k_{\mathrm{ion}}\;□\;★$	B	−%	−%	−%
$k_{\mathrm{stem}}\;□\;★$	B	0.1%	0.1%	0.1%
$k_{\mathrm{other}}\;□\;★$	B	0.1%	0.1%	0.1%
Statistical $k_{\mathrm{pos}}$ (2 dets) □★	A	−	−	−
Geometry $k_{\mathrm{pos}}\;□\;★$	B	−	−	−
${\left(M_{\mathrm{raw}}\right)}_{f_{\mathrm{clin}},f_{\mathrm{msr}}}$ (single det.)				
Positioning system/FS center $w_{x}$/$w_{y}$	B		0.1/0.1 mm	
Positioning system/FS center	B	0.02%	0.01%	−
Readout/Machine reproducibility	A	0.1%	0.1%	0.1%
Jaws reproducibility ${\mathrm{FS}}_{x}$/${\mathrm{FS}}_{y}$	A		0.02/0.05 mm	
Jaws reproducibility $S_{\mathrm{clin}}$	A	0.026 mm (0.32%)	0.027 mm (0.08%)	0.027 mm (0.02%)
Combined uncertainty (k=1)		0.34%	0.13%	0.10%
Field size determination				
Statistical ${\mathrm{FS}}_{x}$/${\mathrm{FS}}_{y}$ (1 centering)	A		0.06/0.07 mm	
Statistical ${\mathrm{FS}}_{x}$/${\mathrm{FS}}_{y}$ (2 centerings)	A		0.04/0.05 mm	
Inter‐det‐type ${\mathrm{FS}}_{x}$/${\mathrm{FS}}_{y}$	B		0.07/0.08 mm	
$S_{\mathrm{clin}}$ (1 centering)□	A	0.071 mm (0.85%)	0.071 mm (0.21%)	0.071 mm (0.04%)
$S_{\mathrm{clin}}$ (2 centerings)★	A	0.062 mm (0.75%)	0.062 mm (0.18%)	0.062 mm (0.04%)
Combined uncertainty (k=1) □		1.75%	0.74%	0.53%
Combined uncertainty (k=1) ★		1.08%	0.61%	0.52%

*Note*: Sources of uncertainty of $M_{f_{\mathrm{clin}},f_{\mathrm{msr}}}$ ${\left(M_{\mathrm{raw}}\right)}_{f_{\mathrm{clin}},f_{\mathrm{msr}}}$ for a single detector and field determination are explicitly detailed. The ★ denotes uncertainties from two detectors with four measurements each, and the □ indicates uncertainty from a single measurement with one detector.

**TABLE 7:** Uncertainty budget example for $\Omega_{Q_{\mathrm{clin}},Q_{\mathrm{msr}}}^{f_{\mathrm{clin}},f_{\mathrm{msr}}}$ measurements with the PTW60019 detector.

Item	Type	Field size [${\mathrm{cm}}^{2}$]		
		0.6 × 0.6	1 × 1	2 × 2
$k_{Q_{\mathrm{clin}},Q_{\mathrm{msr}}}^{f_{\mathrm{clin}},f_{\mathrm{msr}}}$ PTW60019 (see Table 6) □★	B	1.08%	0.61%	0.52%
$M_{f_{clin},f_{\mathrm{msr}}}$ PTW60019				
Statistical $M_{raw}$ (1 meas) □	A	1.3%	0.42%	0.10%
Statistical $M_{raw}$ (2 dets × 4 meas) ★	A	0.46%	0.15%	0.04%
$k_{\mathrm{ion}}\;□\;★$	B	−%	−%	−%
$k_{stem}\;□\;★$	B	0.1%	0.1%	0.1%
$k_{other}\;□\;★$	B	0.1%	0.1%	0.1%
Statistical $k_{pos}$ (2 dets) □★	A	−	−	−
Geometry $k_{pos}\;□\;★$	B	−	−	−
Field size determination				
$S_{clin}$ (1 centering) □★	A	0.85%	0.21%	0.04%
Combined uncertainty (k=1) □		1.90%	0.78%	0.55%
Combined uncertainty (k=1) ★		1.46%	0.68%	0.54%

*Note*: The sources of uncertainty of PTW60019 $k_{Q_{\mathrm{clin}},Q_{\mathrm{msr}}}^{f_{\mathrm{clin}},f_{\mathrm{msr}}}$, $M_{f_{\mathrm{clin}},f_{\mathrm{msr}}}$, and field determination are explicitly detailed. The ★ denotes uncertainties from two detectors with four measurements each, and the □ indicates uncertainty from a single measurement with one detector.

### 3.4 $k_{Q_{\mathrm{clin}},Q_{\mathrm{msr}}}^{f_{\mathrm{clin}},f_{\mathrm{msr}}}$ of all detectors

**FIGURE 4:**
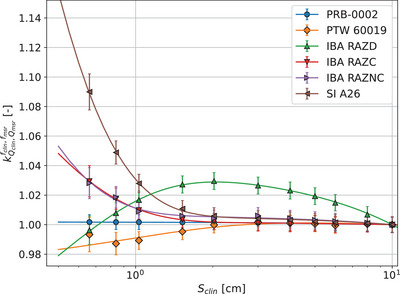
Extracted output correction factors $k_{Q_{\mathrm{clin}},Q_{\mathrm{msr}}}^{f_{\mathrm{clin}},f_{\mathrm{msr}}}$ for different detectors as a function of field size. The fit combines a modified sigmoidal component and a linear adjustment to model both saturating behavior and subsequent linear trends.

Field output correction factors evaluated in this study for all detectors irradiated with 6 MV Varian Truebeam fields of field sizes in the range of 0.6 × 0.6 $\;cm^{2}$ to 40 × 40 $\;cm^{2}$ are presented in Figure 4 and Table 8. Comparisons with published values are shown in Supplementary material Section 4. Considering uncertainties, $k_{Q_{\mathrm{clin}},Q_{\mathrm{msr}}}^{f_{\mathrm{clin}},f_{\mathrm{msr}}}$ of PRB‐0002 is the closest to unity of all studied detectors. Uncertainties on correction factors obtained with the PRB‐0002 are lower than those listed in TRS‐483^4^ and those from Casar et al.^6^, ^8^ for other detectors. Correction factors remain small (<1.5%) over the whole set of investigated field sizes for the PTW microDiamond. IBA Razor diode exhibits known higher correction factors due to an increased response to lower energy photons present in larger fields. Due to the msr field normalization, IBA RAZD field output correction factors range from +3% for 2 × 2 $\;cm^{2}$ down to −10% at 40 × 40 $\;cm^{2}$. Micro chambers exhibit a non‐negligible correction factor (>1%) for field sizes of 1 × 1 $\;cm^{2}$ and below.

**TABLE 8:** Extracted field output correction factors for several detectors used in this study.

Field size (cm)		$k_{Q_{\mathrm{clin}},Q_{\mathrm{msr}}}^{f_{\mathrm{clin}},f_{\mathrm{msr}}}$ (−)					
Nominal	Measured	PRB‐0002	PTW 60019	IBA RAZD	IBA RAZC	IBA RAZNC	SI A26
0.60	0.66	1.002 (5)	0.993 (11)	0.996 (9)	1.029 (10)	1.028 (10)	1.090 (12)
0.80	0.84	1.002 (5)	0.987 (7)	1.008 (6)	1.018 (7)	1.019 (7)	1.049 (7)
1.00	1.03	1.002 (5)	0.989 (6)	1.017 (6)	1.010 (6)	1.009 (6)	1.028 (6)
1.50	1.52	1.002 (5)	0.995 (5)	1.027 (5)	1.004 (5)	1.006 (5)	1.011 (5)
2.00	2.02	1.001 (5)	1.000 (5)	1.030 (5)	1.002 (5)	1.004 (5)	1.006 (5)
3.00	3.00	1.001 (5)	1.002 (5)	1.026 (5)	1.001 (5)	1.006 (5)	1.004 (5)
4.00	4.00	1.001 (5)	1.001 (5)	1.023 (5)	1.001 (5)	1.004 (5)	1.003 (5)
5.00	5.00	1.001 (5)	1.001 (5)	1.019 (5)	1.001 (5)	1.003 (5)	1.002 (5)
6.00	6.00	1.001 (4)	1.000 (5)	1.015 (5)	1.000 (5)	1.002 (5)	1.001 (5)
8.00	8.00	1.000 (5)	1.000 (5)	1.007 (5)	1.000 (5)	1.001 (5)	1.001 (5)
10.00	10.00	1.000 (5)	1.000 (5)	1.000 (5)	1.000 (5)	1.000 (5)	1.000 (5)
20.00	20.00	0.999 (5)	0.998 (5)	0.960 (5)	1.000 (5)	0.999 (5)	1.000 (5)
30.00	30.00	0.999 (5)	1.000 (6)	0.928 (6)	1.003 (6)	1.000 (6)	1.002 (6)
40.00	40.00	1.000 (6)	1.002 (6)	0.906 (6)	1.007 (6)	1.004 (6)	1.006 (6)

*Note*: Uncertainties are shown in brackets and represent absolute uncertainties in the last or two last digits.

Comparison of $k_{Q_{\mathrm{clin}},Q_{\mathrm{msr}}}^{f_{\mathrm{clin}},f_{\mathrm{msr}}}$ with published data from TRS‐483^4^ shows only a statistically significant difference for the PTW microDiamond at field sizes of 1 × 1 $\;cm^{2}$ and below. Since published data from Casar et al.^6^, ^8^ applies specifically to a total correction factor and not directly to $k_{Q_{\mathrm{clin}},Q_{\mathrm{msr}}}^{f_{\mathrm{clin}},f_{\mathrm{msr}}}$, we divided their reported total correction factor by our interpolated $k_{\mathrm{pol}}$ and $k_{\mathrm{ion}}$ ratios between clin and msr fields, in order to be able to compare $k_{Q_{\mathrm{clin}},Q_{\mathrm{msr}}}^{f_{\mathrm{clin}},f_{\mathrm{msr}}}$ values with the same definition. No statistically significant differences were then observed on the whole field size range. Comparison with published data from Gul et al.^39^ and Looe et al.^38^ show however important differences for field output correction factors of IBA Razor diodes and Nano Chambers at small field sizes.

## 4 DISCUSSION

RT is increasingly reliant on small radiation fields to deliver highly conformal treatments. To take full advantage of these complex dose distributions, small fields must be accurately modeled. However, measurements in these conditions are challenging because of the changes with field size in the dose to detector versus dose to water distribution, as well as changes in detector response with either operating conditions (polarity for example) or changing particle energy spectra and flux. Reports detailing advances in small field dosimetry have recently been published^4^, ^5^ to offer guidance and data in order to help define a uniform practice.

In parallel to these efforts in improving the practice of small field dosimetry, equipment manufacturers are also developing new instruments better suited to measure small radiation fields. One such example is Medscint's PRB‐0002 studied in this work. This detector was designed specifically to minimize the correction factors needed when measuring output factors of small fields. As seen of Figure 3, perturbations caused by the density and atomic composition of the scintillator, $P_{\mathrm{scint}}$ is nearly entirely balanced by changes in ionization quenching, $k_{\mathrm{ioq}}$, therefore providing a $k_{Q_{\mathrm{clin}},Q_{\mathrm{msr}}}^{f_{\mathrm{clin}},f_{\mathrm{msr}}}$ close to unity for the entire range of field sizes considered. Minimizing the amplitude of correction factors that must be applied to obtain the dose in water from a detector's reading makes this process less sensitive to errors, therefore simplifying both the experimental procedure and dose calculation itself.

A careful uncertainty analysis is critical for characterization of new detectors for a meaningful, quantitative comparison with existing detectors. Throughout this work efforts were made to keep uncertainty as small as possible. A detailed uncertainty budget was provided that included: averaging measurements from multiple detectors of the same model, using an 11‐points positioning technique, evaluation of jaw reproducibility and sensitivity assessment for analytical and MC calculations. It is important to note that, at the smallest field size, the largest source of uncertainty comes from the field size determination.

The detailed uncertainty characterization makes it possible to identify small factors of influence that could otherwise be attributed to stochastic fluctuations. One such factor for PSD is quenching. While ionization quenching in scintillators has been known for decades,^40^ it was generally assumed to be negligible for megavoltage photon beams. However, Santurio and Andersen recently showed that the contribution of low‐energy secondary electrons is sufficient to cause quenching; furthermore, the changing spectrum of secondary electrons with field size causes the quenching effect to be dependent on field size.^13^ This means that $k_{Q_{\mathrm{clin}},Q_{\mathrm{msr}}}^{f_{\mathrm{clin}},f_{\mathrm{msr}}}$ for PSD may be farther from unity than initially claimed.^4^ This work is the first to quantify $k_{\mathrm{ioq}}$ for a commercial PSD and to show how this quantification can help design a detector with a $k_{Q_{\mathrm{clin}},Q_{\mathrm{msr}}}^{f_{\mathrm{clin}},f_{\mathrm{msr}}}$ as close to unity as possible. In the case of the PRB‐0002 detector, the change of $k_{\mathrm{ioq}}$ with field size is mostly compensated by an inverse change of $P_{\mathrm{scint}}$ as seen on Figure 3

While great care was taken to express our findings within the framework established by guiding documents,^4^, ^5^ it was not entirely possible to do so. In this work we've explicitly differentiated two broad categories of correction for factors of influence: type I and type II. Both types represent corrections that must be applied to a *raw* detector reading so that the corrected values can be assumed to be proportional to the average dose deposited in the sensitive volume of a detector (see Equations 5 and 6). Type I factors are assumed to be independent of small beam quality changes that can occur between a reference field and a measured field (i.e. going from $Q_{\mathrm{msr}}$ to $Q_{\mathrm{clin}}$ does not change $k_{I}$ factors). They are associated with known external factors of influence (e.g., $k_{TP}$), that can also change with field size (e.g., $k_{\mathrm{ion}}$, $k_{\mathrm{pol}}$), but not directly because of a beam quality change. Following TRS‐483, they are applied to raw measurement before the field output correction factor. With type II factors, small changes in beam quality occurring when going from $f_{\mathrm{msr}}$ to $f_{\mathrm{clin}}$ is considered within the cause of intrinsic detector response variation. Because these factors do not involve an influencing quantity that is externally measurable and easily factorizable, they are herein proposed to be included in the field output correction factor. Traditionally, all corrections were assumed to be independent of beam quality changes and thus fell in the type I category. We believe the distinction between categories I and II is important to be made explicit even if, for now, it mainly concerns PSD detectors. Other corrections factors could be classified as type II. For example, a part of $k_{\mathrm{ion}}$ ratio is attributed to initial recombination, which is beam quality dependent and could be seen as type II. With the proposed equation for $k_{\mathrm{ion}}$ ratio correction (see Equation 12), it is assumed that the $k_{\mathrm{ion}}$ change with field size is only dependent on the dose per pulse variation. Therefore, if there is any change in initial recombination rate with field size, this will be included in the field output correction factor. However, knowing the rather small importance of general ion recombination variations for IC and SS detectors in the dose per pulse range of standard electron/photon external beam radiotherapy (see Table 3), it can be expected that the remaining initial recombination variation will be negligible.

Using Equation (17), field output correction factors for the new PRB‐0002 were determined with an uncertainty of about 0.5% (see Table 4 for details). This precision is comparable or better than $k_{Q_{\mathrm{clin}},Q_{\mathrm{msr}}}^{f_{\mathrm{clin}},f_{\mathrm{msr}}}$ uncertainties of other detectors listed in TRS‐483.^4^ Furthermore, using this detector to measure output factors (see Table 5) may lead to more precise measurements than those listed in references.^4^, ^6^ Mostly excellent agreement is seen in the comparison of field output correction factors of different detectors to published data for the same type of linear accelerator and detector orientation.^4^, ^6^, ^8^, ^41^, ^42^ However, care should be taken to follow the same field output correction factor definition. For example, Casar et al. implicitly included the ion recombination and polarity effects in the correction factor. This had to be factored out in order to compare $k_{Q_{\mathrm{clin}},Q_{\mathrm{msr}}}^{f_{\mathrm{clin}},f_{\mathrm{msr}}}$ values following TRS‐483 definition. It is to be noted that, as others have reported (see comments on TRS‐483 by Das and refs therein^43^ and following reply^44^), we measured a small turnaround of $k_{Q_{\mathrm{clin}},Q_{\mathrm{msr}}}^{f_{\mathrm{clin}},f_{\mathrm{msr}}}$ for PTW 60019 at the smallest field size that is significantly different than what is reported in TRS‐483. This effect arises at the low end of the region where volume averaging is partially countered by the density effect of diamond over‐response that changes when field size approaches detector dimensions.^45^ However, it could also involve radiation‐induced charge imbalance created in the connections below the sensitive volume.^46^ The reason why some studies show this feature and others don't is still not definitively answered. Another statistically significant difference observed was between our measured small fields $k_{Q_{\mathrm{clin}},Q_{\mathrm{msr}}}^{f_{\mathrm{clin}},f_{\mathrm{msr}}}$ factors for the newer IBA Razor Nano chamber and the ones reported by Gul et al.^39^ and Looe et al.^38^ (see Table S8). Despite both correcting for polarity effect, they observe a lower field output correction factor. The fact that the $k_{Q_{\mathrm{clin}},Q_{\mathrm{msr}}}^{f_{\mathrm{clin}},f_{\mathrm{msr}}}$ factor reported by Gul et al. for the IBA Razor diode is also lower might indicate a difference in their reference detector output ratio. A much better agreement for the IBA Razor Nano chamber is seen with recent Mateus et al.^42^ data which uses a TRS‐483 corrected PTW 60019 detector as reference and with data reported by Girardi et al.^47^ with Gafchromic EBT3 films as reference. These differences between studies seen with the smallest volume micro IC might also need further investigations.

Measuring precise field output factors of small radiation fields can be a rewarding process when modeling beam source parameters for a TPS, as it will impact the quality of dose calculations for simple small target cases or more complex modulated treatment techniques. However, great care should be deployed in measuring and applying correction factors. Also understanding the physical effects involved in $k_{Q_{\mathrm{clin}},Q_{\mathrm{msr}}}^{f_{\mathrm{clin}},f_{\mathrm{msr}}}$ at small fields is important to better asses uncertainties and potential zones of detector development. As examples, Table S4 Section 3 illustrates the importance of volume averaging effects following the choice of detector and orientation, and MC studies separating cameral, sensitive material and density, as well as recombination and signal collection effects, are useful in tackling detector design issues and new opportunities. However, given the work involved in determining these correction factors, day‐to‐day clinical practice can be simplified by selecting a detector that required only minimal corrections.

With PRB‐0002, taking advantage of having a $k_{Q_{\mathrm{clin}},Q_{\mathrm{msr}}}^{f_{\mathrm{clin}},f_{\mathrm{msr}}}$ close to unity with small and well characterized uncertainties, we used Equation (4) to determine the $k_{Q_{\mathrm{clin}},Q_{\mathrm{msr}}}^{f_{\mathrm{clin}},f_{\mathrm{msr}}}$ of other detectors and then used these detectors to measure $\Omega_{Q_{\mathrm{clin}},Q_{\mathrm{msr}}}^{f_{\mathrm{clin}},f_{\mathrm{msr}}}$. For example, applying this process to the PTW microDiamond, uncertainties between 1.9% (0.6×0.6 ${\mathrm{cm}}^{2}$ field) and 0.55% (2×2 ${\mathrm{cm}}^{2}$ field) can be reached for a single measurement.

## 5 CONCLUSION

In this work we first present a detailed determination of field output correction factor, $k_{Q_{\mathrm{clin}},Q_{\mathrm{msr}}}^{f_{\mathrm{clin}},f_{\mathrm{msr}}}$ for the new PRB‐0002; then we used that value to determine the detector specific field output correction factors for a large number of detectors, both ICs and SS detectors. Our work complements and validates the results from other publications such as on $k_{\mathrm{pol}}$ ^38^ and $k_{Q_{\mathrm{clin}},Q_{\mathrm{msr}}}^{f_{\mathrm{clin}},f_{\mathrm{msr}}}$.^4^, ^6^, ^39^ We carefully considered the causes and magnitude of uncertainties and provided realistic measurement scenarios with uncertainty budgets (Tables 4, 5, 6, 7) to help others in the challenging task of accurate dose measurement in small fields.

Ionization quenching in PSDs has been shown to have a measurable impact in megavoltage photon beams when going from small to large fields.^13^ This is explained by the increased amount of low energy scatter radiation as field size becomes larger. Low energy radiation causes more quenching, which is reflected in the increase of $k_{\mathrm{ioq}}$ with field size. The change of energy spectra with field size also impact $P_{\mathrm{scint}}$, the correction factor accounting for perturbations due to the variation in density and atomic composition of the scintillating material compared to water. Our work is the first to show that for the new PRB‐0002, the impact of $k_{\mathrm{ioq}}$ and $P_{\mathrm{scint}}$ compensate each other to produce a $k_{Q_{\mathrm{clin}},Q_{\mathrm{msr}}}^{f_{\mathrm{clin}},f_{\mathrm{msr}}}$ close to 1 for all field sizes.

## CONFLICT OF INTEREST STATEMENT

Some authors (B.C., S.L., D.L., Y.L., F.T.) are employees of Medscint, which produces one of the products discussed in this paper. They participated in the data acquisition and Monte Carlo simulation components of the study; however, the research and conclusions presented were conducted independently and are not influenced by any commercial interests.

## Supporting information

Supporting Information
