# Supplementary material for: Field output correction factors using a scintillation detector
Source: Med Phys. 2025 Mar 8;52(6):4844–61. doi: 10.1002/mp.17729 (PMC12149716; doi:10.1002/mp.17729)
Supplement: Supplementary file 1 — Supporting Information [file MP-52-4844-s001.pdf]

Supplementary material

1 Definitions of k_I corrections

- k_{TP}: the linear temperature and pressure corrections normalizing ionization chamber environmental response to standard reference condition (22 °C, 101.33 kPa)[16].
- k_{H}: air humidity correction factor. For operations in the range of 20-80% relative humidity, k_{H} may be considered as unity with a 0.15% uncertainty [16].
- k_{T}: temperature correction factor normalizing SS and PSD detector response to standard reference condition of 22 °C.
- k_{elec}: electrometer correction factor to correctly account for electric current produced in the detector.
- k_{read}: readout correction for signals from devices other than an electrometer. For PSDs, it is the conversion factor from raw digital readout of the optical sensor back to the light output of the detector. It can change with time and can be a result of sensor thermal drift or optical coupling variations.
- $k_{\mathrm{pol}}^{+/-}$: ion chamber polarity correction factor that averages charge accumulated at both positive and negative polarity and is applied to signal acquired with either + or − polarity.
- k_{ion}: ionization recombination correction factor for ionization chambers that depends on the detector operation polarizing voltage [16]. For SS detectors this is the incomplete charge collection factor that accounts for change in sensitivity and dose rate dependence due to recombination-generation centres and traps included in the crystalline structure of the sensitive volume.
- k_{drift}: correction for machine output drifts with time.
- k_{bg}: correction for radiation dose contribution that do not come from an intended irradiation from the dose delivery apparatus. This correction factor should be unity if all background contributions are correctly subtracted from the measured raw signal.

- k_{pos}: detector position uncertainty correction factor. M^{det} is intended to be the measurement corrected for all influence quantities, other than beam quality, but including detector positional uncertainty. This means that this is the value obtained with perfect detector positioning. This factor therefore correct for impact of position uncertainty. See section II.C.2 for more details.

- k_{stem}: this correction factor is intended to remove any contribution to the raw measurement that come from irradiation of any part of the detector system other than the sensitive volume. Along with k_{bg}, this is the second correction needed for signal leakage removal.

- k_{other}: any other correction factor

2 Determination of detectors geometrical and positional response to dose distribution

Detector sensitive volume geometries may be either spherical, with a radius r; cylindrical, with a radius r and length l; or cylindrical with a half-spherical tip, with a cylindrical length l and common sphere and cylinder radius r. Two orientations were considered for cylindrical detectors, with either their symmetry axis parallel or perpendicular to the beam axis (along z or y direction respectively).

Given these geometries, the integral in eq. (21) is performed either in a Polar or Cartesian coordinate system, with the coordinate transformations $x - x_0 = \rho \cos\theta$ and $y - y_0 = \rho \sin\theta$. Table 1 presents the coordinate system, analytical function h, and cross sectional area A used for each combination of geometry and orientation. Table 2 gives the functions $M(x_0, y_0)$ obtained from the integration of eq. (21). The expected normalized measurement signal functions, $\langle M(x_0, y_0)\rangle$, obtained from eq. (22) using the equations from Table 2 are given in Table 3.

Table 1: Detector height function and cross-sectional area used in equation (21).

Geometry	Orientation	Coord. System	h	A
Spherical	-	Polar	$\frac{3}{2\pi r^3}\sqrt{r^2-\rho^2}$	$\int_0^r \int_0^{2\pi} \rho d\theta d\rho$
Cylindrical	$\parallel$ z	Polar	$\frac{1}{\pi r^2}$	$\int_0^r \int_0^{2\pi} \rho d\theta d\rho$
Cylindrical	$\perp$ y	Cartesian	$\frac{2}{\pi r^2 l}\sqrt{r^2-(x-x_0)^2}$	$\int_{-l/2}^{l/2} \int_{-r}^{r} dx dy$
Cyl / Half-Sph	$\parallel$ z	Polar	$\frac{1}{\pi r^2(l+2r/3)}(l+\sqrt{r^2-\rho^2})$	$\int_0^r \int_0^{2\pi} \rho d\theta d\rho$

3 Impact of the measured profiles on k_{vol} for PRB-0002

Table 4 shows *kvol* correction factors for each detector determined with their respective fitted profiles and appropriate geometrical functions (see Eq. 20 to 24

Table 2: Analytical formulation of normalized measurement signal described by eq. (21) at position (x_0, y_0) for different detector geometry and orientation.

Geometry	Orientation	$M(x_0, y_0)$
Spherical	-	$(f(x_0) + \frac{a_2 r^2}{5})(g(y_0) + \frac{b_2 r^2}{5}) - \frac{a_2 b_2 r^4}{70}$
Cylindrical	$\parallel$ z	$(f(x_0) + \frac{a_2 r^2}{4})(g(y_0) + \frac{b_2 r^2}{4}) - \frac{a_2 b_2 r^4}{48}$
Cylindrical	$\perp$ y	$(f(x_0) + \frac{a_2 r^2}{4})(g(y_0) + \frac{b_2 r^2}{12})$
Cyl / Half-Sph	$\parallel$ z	$\frac{2r/3}{l+2r/3} M_{sph}(x_0, y_0) + \frac{l}{l+2r/3} M_{cyl\parallel}(x_0, y_0)$

Table 3: Analytical formulation of expected normalized measurement signal, M, given by eq. (22) at position (x_0, y_0) for different detector geometries and orientations.

Geometry	Orientation	$\langle M(x_0, y_0)\rangle$
Spherical	-	$M(x_0, y_0) + (f(x_0) + \frac{a_2 r^2}{5})\frac{b_2 w_y^2}{3} + (g(y_0) + \frac{b_2 r^2}{5})\frac{a_2 w_x^2}{3} + \frac{a_2 b_2 w_x^2 w_y^2}{9}$
Cylindrical	$\parallel$ z	$M(x_0, y_0) + (f(x_0) + \frac{a_2 r^2}{4})\frac{b_2 w_y^2}{3} + (g(y_0) + \frac{b_2 r^2}{4})\frac{a_2 w_x^2}{3} + \frac{a_2 b_2 w_x^2 w_y^2}{9}$
Cylindrical	$\perp$ y	$M(x_0, y_0) + (f(x_0) + \frac{a_2 r^2}{4})\frac{b_2 w_y^2}{3} + (g(y_0) + \frac{b_2 r^2}{12})\frac{a_2 w_x^2}{3} + \frac{a_2 b_2 w_x^2 w_y^2}{9}$
Cyl / Half-Sph	$\parallel$ z	$\frac{2r/3}{l+2r/3} \langle M_{sph}\rangle (x_0, y_0) + \frac{l}{l+2r/3} \langle M_{cyl\parallel}\rangle (x_0, y_0)$

and Supplementary material section 3). Because profiles can be measured with any detector, Table 5 compares impact of using different detectors for profile measurements to determine k_{vol}. The average value was used to determine $k_{Q_{\mathrm{clin}},Q_{\mathrm{msr}}}^{f_{\mathrm{clin}},f_{\mathrm{msr}}}$ of the PRB-0002.

Table 4: Extracted volume component of field output correction factors. All detectors aligned parallel to beam axis, except PRB-0002 which is perpendicular along the inline axis.

Field Size (cm)		k_{vol} (-)					
Nominal	Measured	PRB-0002 ($\perp$)	PTW 60019	IBA RAZD	IBA RAZC	IBA RAZNC	SI A26
0.50	0.54	-	1.033	1.003	1.026	1.025	-
0.60	0.64	1.006	1.023	1.002	1.021	1.017	1.040
0.80	0.82	1.003	1.012	1.001	1.011	1.012	1.028
1.00	1.00	1.001	1.007	1.000	1.005	1.005	1.014
2.00	1.99	-	1.000	1.000	1.001	1.000	1.001

4 Comparisons of $k_{Q_{\mathrm{clin}},Q_{\mathrm{msr}}}^{f_{\mathrm{clin}},f_{\mathrm{msr}}}$ with published values

Values for $k_{Q_{\mathrm{clin}},Q_{\mathrm{msr}}}^{f_{\mathrm{clin}},f_{\mathrm{msr}}}$ obtained in this study are compared with other published values. Table 6 shows the comparison with values from the TRS-483 report [4], Table 7 compares with the works of Casar et al.[6, 8] and Table 8 compares with Gul et al.[39], Looe et al. [38] and Mateus et al. [42]. In these tables, values of $k_{Q_{\mathrm{clin}},Q_{\mathrm{msr}}}^{f_{\mathrm{clin}},f_{\mathrm{msr}}}$ from this study are extracted from our fit equation at the same reported S_{clin} field size as the compared literature data. Please note that for

Table 5: k_{vol} for PRB-0002 (both $\perp$ and $\|$ to the beam axis) determined using dose profiles measured with different detectors. All detectors used for profile measurement were $\|$ to the beam axis

Field Size (cm)		k_{vol}^{PSD} (-)													
		PRB-0002		PTW 60019		IBA RAZD		IBA RAZC		IBA RAZNC		SI A26		Average	
Nominal	Measured	$\|$	$\perp$	$\|$	$\perp$	$\|$	$\perp$	$\|$	$\perp$	$\|$	$\perp$	$\|$	$\perp$	$\|$	$\perp$
0.50	0.54	-	-	1.0066 (6)	1.0077 (4)	1.0073 (7)	1.0085 (5)	1.0076 (7)	1.0089 (5)	1.0078 (8)	1.0091 (5)	-	-	1.0073 (3)	1.0086 (2)
0.60	0.64	1.0050 (5)	1.0058 (2)	1.0047 (4)	1.0054 (2)	1.0047 (4)	1.0055 (3)	1.0063 (6)	1.0073 (3)	1.0052 (5)	1.0060 (3)	1.0045 (4)	1.0052 (2)	1.0050 (2)	1.0059 (1)
0.80	0.82	1.0022 (2)	1.0026 (1)	1.0025 (2)	1.0029 (1)	1.0025 (2)	1.0030 (1)	1.0032 (3)	1.0037 (2)	1.0038 (3)	1.0044 (2)	1.0031 (3)	1.0036 (1)	1.0029 (1)	1.0034 (0)
1.00	1.00	1.0011 (1)	1.0013 (0)	1.0013 (1)	1.0016 (0)	1.0012 (1)	1.0014 (0)	1.0016 (1)	1.0019 (1)	1.0016 (1)	1.0019 (0)	1.0016 (1)	1.0019 (0)	1.0014 (0)	1.0017 (0)
2.00	1.99	-	-	1.0001 (0)	1.0001 (0)	1.0001 (0)	1.0001 (0)	1.0002 (0)	1.0002 (0)	1.0001 (0)	1.0002 (0)	1.0001 (0)	1.0001 (0)	1.0001 (0)	1.0001 (0)

TRS-483 the $k_{Q_{\text{clin}},Q_{\text{msr}}}^{f_{\text{clin}},f_{\text{msr}}}$ values for ion chambers are tabulated for perpendicular orientations while our ion chamber measurements were done with parallel orientations. This should not impact A26 which is spherical, but could have a minor effect on the RAZC at smallest fields.

Table 6: Comparison of field output correction factors from this study and values from IAEA TRS-483 [4] for several detectors. Uncertainties are shown in brackets and represent absolute uncertainties in the last or two last digits.

	$k_{Q_{\text{clin}},Q_{\text{msr}}}^{f_{\text{clin}},f_{\text{msr}}}$ (-)											
	PTW 60019			IBA RAZD/SFD			IBA RAZC/CC01			SI A26		
Field Size (cm)	this study	TRS-483	p-value	this study	TRS-483	p-value	this study	TRS-483	p-value	this study	TRS-483	p-value
0.60	0.985 (8)	0.968 (7)	0.001	0.990 (11)	0.990 (6)	0.998	1.035 (14)	1.047 (26)	0.391	1.110 (16)	1.165 (29)	0.188
0.80	0.988 (6)	0.977 (5)	0.002	1.006 (6)	1.007 (6)	0.747	1.019 (7)	1.027 (16)	0.352	1.056 (7)	1.062 (16)	0.714
1.00	0.991 (5)	0.984 (4)	0.014	1.016 (5)	1.018 (5)	0.446	1.011 (5)	1.018 (11)	0.230	1.031 (5)	1.023 (11)	0.502
1.50	0.996 (4)	0.993 (4)	0.276	1.027 (4)	1.030 (5)	0.227	1.003 (4)	1.011 (6)	0.061	1.010 (4)	1.003 (6)	0.324
2.00	0.998 (4)	0.997 (3)	0.574	1.029 (5)	1.032 (4)	0.243	1.002 (4)	1.009 (4)	0.055	1.006 (5)	1.000 (4)	0.248
3.00	1.000 (4)	1.000 (4)	0.821	1.027 (5)	1.029 (4)	0.395	1.001 (5)	1.008 (4)	0.069	1.004 (5)	1.000 (4)	0.361
4.00	1.001 (4)	1.000 (3)	0.569	1.023 (4)	1.025 (3)	0.407	1.001 (4)	1.007 (4)	0.082	1.004 (4)	1.000 (4)	0.415
5.00	1.001 (4)	1.000 (3)	0.522	1.019 (4)	1.021 (3)	0.390	1.001 (4)	1.006 (4)	0.121	1.003 (3)	1.000 (4)	0.478
6.00	1.001 (3)	1.000 (3)	0.533	1.015 (3)	1.017 (3)	0.357	1.001 (3)	1.004 (3)	0.159	1.002 (2)	1.000 (3)	0.455
8.00	1.001 (1)	1.000 (3)	0.654	1.008 (1)	1.008 (3)	0.792	1.000 (1)	1.002 (3)	0.299	1.001 (1)	1.000 (3)	0.677
10.00	1.000 (0)	1.000 (0)	1.000	1.000 (0)	1.000 (0)	1.000	1.000 (0)	1.000 (0)	1.000	1.000 (0)	1.000 (0)	1.000

Table 7: Comparison of field output correction factors from this study and values from Casar et al. [6, 8] for three detectors oriented parallel to the beam axis. (∗): Values from reference, but modified to exclude expected k_{pol} and k_{ion} factors. Uncertainties are shown in brackets and represent absolute uncertainties in the last or two last digits.

	$k^{f_{\mathrm{clin}},f_{\mathrm{msr}}}_{Q_{\mathrm{clin}},Q_{\mathrm{msr}}}$ (-)								
	PTW 60019			IBA RAZD			IBA RAZC		
Field Size (cm)	this study	Casar et al. [6]*	p-value	this study	Casar et al. [6]*	p-value	this study	Casar et al. [8]*	p-value
0.56	0.984 (9)	0.976 (19)	0.515	0.986 (13)	0.991 (19)	0.731	1.040 (17)	1.063 (22)	0.217
0.81	0.989 (6)	0.970 (13)	0.122	1.007 (6)	1.000 (13)	0.494	1.019 (6)	1.018 (14)	0.937
1.01	0.991 (5)	0.985 (12)	0.488	1.016 (5)	1.018 (12)	0.794	1.011 (5)	1.016 (12)	0.529
1.50	0.996 (4)	0.992 (11)	0.669	1.027 (4)	1.023 (11)	0.595	1.003 (4)	1.004 (11)	0.966
2.00	0.998 (4)	0.995 (11)	0.712	1.029 (5)	1.023 (12)	0.479	1.002 (4)	0.999 (12)	0.760
3.03	1.001 (4)	1.000 (11)	0.896	1.027 (5)	1.024 (11)	0.733	1.001 (5)	1.002 (11)	0.879
4.03	1.001 (4)	0.999 (11)	0.741	1.023 (4)	1.019 (11)	0.627	1.001 (4)	1.001 (11)	0.989
5.02	1.001 (4)	0.996 (10)	0.450	1.019 (4)	1.012 (11)	0.401	1.001 (4)	0.997 (10)	0.632
10.03	1.000 (0)	1.001 (0)	0.000	1.000 (0)	1.001 (1)	0.192	1.000 (0)	1.001 (0)	0.000

Table 8: Comparison of field output correction factors from this study and values from Gul et al. [39], Looe et al. [38] and Mateus et al. [42] for two detectors. Uncertainties are shown in brackets and represent absolute uncertainties in the last or two last digits.

	$k^{f_{\mathrm{clin}},f_{\mathrm{msr}}}_{Q_{\mathrm{clin}},Q_{\mathrm{msr}}}$ (-)								
	IBA RAZD			IBA RAZNC					
Field Size (cm)	this study	Gul et al. [39]	p-value	this study	Gul et al. [39]	p-value	Looe et al. [38]	Mateus et al. [42]	p-value
0.52	0.981 (15)	0.948 (2)	0.003	1.049 (21)	0.998 (4)	0.017	0.960 (23)	- (-)	-
0.60	0.990 (11)	- (-)	-	1.036 (13)	- (-)	-	0.962 (23)	1.042 (23)	0.724
0.80	1.006 (6)	- (-)	-	1.019 (7)	- (-)	-	0.980 (23)	1.009 (23)	0.550
1.00	1.016 (5)	1.004 (4)	0.002	1.011 (5)	0.981 (4)	0.000	0.991 (23)	1.005 (23)	0.689
1.50	1.027 (4)	1.019 (3)	0.012	1.006 (4)	0.991 (2)	0.005	0.997 (23)	1.000 (13)	0.502
2.00	1.029 (5)	1.020 (4)	0.011	1.005 (4)	0.996 (3)	0.023	1.006 (23)	- (-)	-
2.51	1.028 (5)	1.023 (3)	0.078	1.005 (4)	0.998 (3)	0.058	- (-)	1.000 (13)	0.586
3.00	1.027 (5)	1.020 (2)	0.027	1.005 (4)	0.998 (2)	0.063	1.001 (23)	1.000 (13)	0.609
4.00	1.023 (4)	1.018 (3)	0.068	1.004 (4)	0.997 (3)	0.040	1.000 (23)	1.000 (13)	0.657
10.00	1.000 (0)	1.000 (4)	1.000	1.000 (0)	1.000 (4)	1.000	- (-)	1.000 (3)	1.000
